# Serial intravital microscopy reveals temporal dynamics of autoreactive germinal centers in the spleen

**DOI:** 10.1016/j.isci.2026.115340

**Published:** 2026-03-11

**Authors:** Layla Pohl, Thomas R. Wittenborn, Ali Shahrokhtash, Kristian S. Kastberg, Cecilia Fahlquist-Hagert, Lisbeth Jensen, Sofie Andkær Pedersen, Julia Karen Demtröder, Donato Sardella, Alain Pulfer, Duncan Sutherland, Santiago F. Gonzalez, Ina Maria Schiessl, Søren E. Degn

**Affiliations:** 1 Department of Biomedicine, Aarhus University, 8000 Aarhus C, Denmark; 2 Interdisciplinary Nanoscience Center (iNANO), Aarhus University, 8000 Aarhus C, Denmark; 3 CellPAT Center for Cellular Signal Patterns, Aarhus University, 8000 Aarhus C, Denmark; 4 Institute for Research in Biomedicine, Faculty of Biomedical Sciences, Università della Svizzera Italiana, 6500 Bellinzona, Ticino, Switzerland; 5 Department of Medicine 2, RWTH Aachen University, 52074 Aachen, Germany

**Keywords:** medical imaging, immunology, cell biology, biological sciences research methodologies

## Abstract

The spleen plays a key role in clearing blood-borne infections and is involved in autoimmune and hematological disorders. It undergoes extensive remodeling during inflammation and immune reactions, but its localization in the peritoneal cavity has hampered studies of these dynamic changes. We establish a protocol for serial two-photon microscopy of the murine spleen to capture dynamic processes in the living animal over weeks. We elucidate the expansion and contraction of autoreactive germinal centers (GCs) induced by the epicutaneous application of the TLR7 agonist resiquimod. Leveraging a biocompatible abdominal imaging window, intravital labeling, and fluorescent reporters, we follow GCs for >2 weeks up to 180 μm below the capsule by tracking follicular dendritic cell networks. We also demonstrate that we can repeatedly image dynamic cell migration in the same area over a two-week period without appreciable perturbation of normal physiology, enabling a deeper understanding of the spleen and its associated disease states.

## Highlights

• Serial two-photon imaging enables longitudinal visualization of the murine spleen
• Biocompatible window allows repeated intravital imaging over 2 weeks
• Autoreactive germinal centers can be tracked during TLR7-driven inflammation
• Dynamic immune cell migration can be visualized without perturbing physiology

## Graphical abstract

**Figure:**
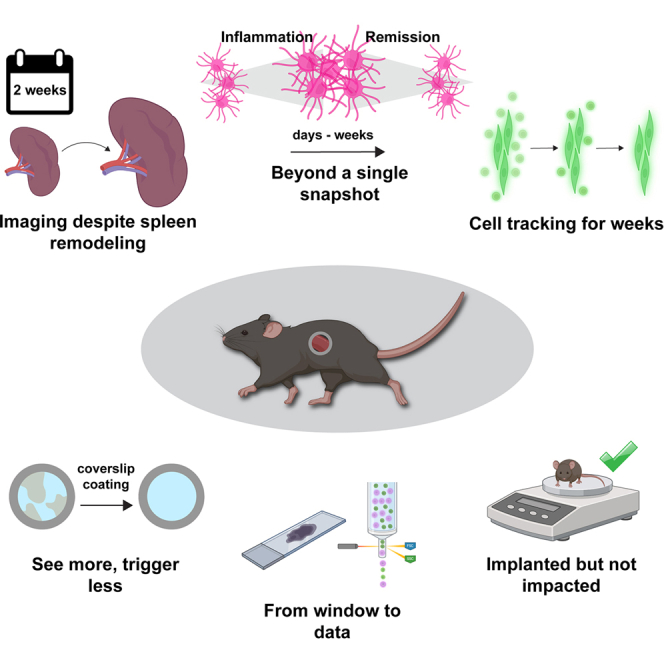

## Introduction

The spleen is the largest secondary lymphoid organ. It harbors two morphologically and functionally distinct compartments, red and white pulp, separated by the marginal zone. In white pulp, B cells are organized in follicles surrounding the periarteriolar lymphoid sheath (PALS), containing dendritic cells and T cells. The red pulp and the marginal zone contain macrophages, which filter out damaged, senescent erythrocytes and foreign material, whereas the white pulp orchestrates adaptive immune responses to captured antigens.^1^ Hence, the spleen plays a key role in clearing blood-borne infections and is involved in numerous systemic conditions, including autoimmune diseases such as systemic lupus erythematosus (SLE), and hematological disorders.^2^

During immune reactions, the spleen undergoes extensive remodeling, but due to its localization in the peritoneal cavity, it has been challenging to study these dynamic changes. Intravital microscopy in mice has elucidated the transport of immune complexes across the marginal zone to underlying follicles,^3^ a key step in antigen presentation, and the influx of T cells along perivascular-T (PT)-tracks during an active immune response.^4^ However, these studies relied on open surgery and were limited to timespans of hours, precluding the longitudinal tracking of dynamic immune processes occurring over days to weeks.

Longitudinal studies of immune processes require intact lymph and blood flow and the absence of untoward inflammation. This was achieved with imaging windows for superficial cutaneous lymph nodes to study cancer metastases,^5^^,^^6^^,^^7^ germinal center (GC) B-cell responses,^8^ and follicular T-cell subsets.^9^ An abdominal imaging window (AIW) was also developed to study physiological processes in internal organs,^10^^,^^11^ and leveraged to interrogate the dynamic function of the kidney,^12^^,^^13^^,^^14^ the pancreas,^15^ and the liver.^16^^,^^17^ The spleen, however, is a particularly challenging organ because of its high blood-flow, thick capsule, high density of active and autofluorescent immune cell populations,^18^ and its ability to dynamically expand and contract over a very large size range.

Here, we present functional studies in the spleen over several weeks using an AIW. We leverage an anti-fouling approach to increase biocompatibility and demonstrate that the AIW does not perturb immune homeostasis. By adoptive transfer of carboxyfluorescein succinimidyl ester (CFSE)-labelled B cells and endogenous reporter fluorescence in CD8^+^ T cells (CD8a-cre;tdTom^flx/flx^), we demonstrate that we can repeatedly image dynamic cell migration in the same splenic area over a two-week period. Utilizing a pharmacological model of SLE based on the epicutaneous application of the small-molecule Toll-like receptor (TLR)-7 agonist resiquimod (R848),^19^ we moreover track follicular dendritic cell (FDC) network dynamics in the context of an autoimmune response over two weeks.

TLR7 is a well-known driver of autoimmune responses,^20^^,^^21^^,^^22^^,^^23^ and a TLR7 gain-of-function mutation causes SLE in humans.^24^ SLE is characterized by the production of affinity-matured autoantibodies, which can originate from both extrafollicular and GC-driven B-cell responses.^25^^,^^26^ While extrafollicular responses are sufficient for the early hallmarks of lupus,^27^ GC responses are thought to be critical to epitope spreading and progression of disease.^28^ Both patients with, and mouse models of, SLE present with spontaneous GCs in the spleen,^29^^,^^30^^,^^31^ and autoreactive GC responses are known to depend upon B-cell-intrinsic TLR7 signaling.^32^^,^^33^^,^^34^ TLR7 sensing by FDCs also promotes GC responses by provision of type I interferon,^35^ which drives autoreactive B-cell activation.^36^^,^^37^

In GCs, B cells undergo affinity maturation through dark and light zone cycling.^38^ In the light zone, they test their affinity for antigen retained by complement receptor 1 (CR1, CD35) and Fc-gamma receptor IIb (FcγRIIb) on FDCs.^39^ B cells that take up antigen and present antigen-derived peptides to T follicular helper cells (T_FH_) are selected for continued expansion and hypermutation or for plasma cell or memory B-cell differentiation. B cells that cannot bind antigen undergo apoptosis and are phagocytosed by highly active tingible-body macrophages (TBMs).^40^

We show that spontaneous autoreactive GCs driven by TLR7 stimulation are highly dynamic. GCs expand in response to TLR7 but do so to widely varying extents at an individual GC level. Moreover, upon the cessation of TLR7 stimulation, GCs contract but again to widely varying extents. Somewhat surprisingly, the phenotype was completely reversible, indicating that although TLR7 is a significant driver of autoreactivity, it is insufficient for a bona fide break of tolerance.

## Results

**Figure 1:**
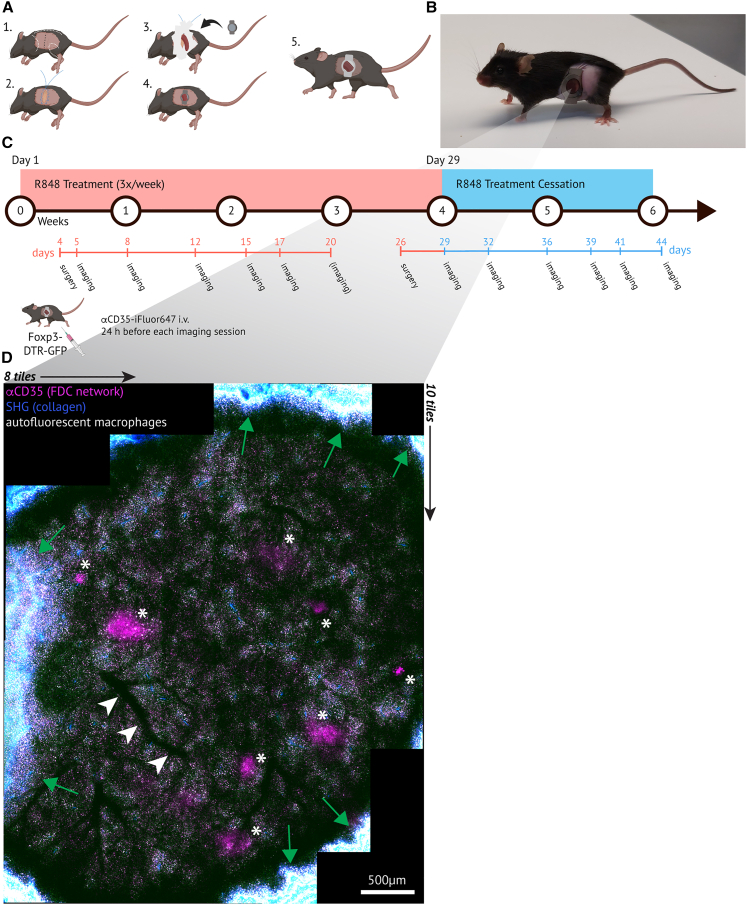
An AIW to track FDC network dynamics in the spleen over 2 weeks using serial intravital microscopy (A) Graphical overview of surgery steps of AIW (abdominal imaging window) implantation: 1. surgical landmarks, 2. purse string suture, 3. spleen mobilization, 4. placement of AIW, and 5. recovered mouse after surgery. (B) Recovered mouse 8 days after AIW implantation. (C) Global treatment timeline: *Foxp3*^DTR−GFP^ mice were treated with R848 three times per week for four weeks to induce an autoimmune response. An AIW was implanted either shortly after R848 treatment initiation or after 4 weeks of treatment. GCs were then followed on six days over 15 days total during or after treatment, respectively. To visualize FDCs in the light zone, mice received αCD35-iFluor647 i.v. 24 h before every imaging session. (D) Maximum intensity projection of a 3D-stitched overview z-stack (100–250 μm below capsule, 150 μm thick) of the entire AIW FOV (8×10 tiles). Asterisks indicate the most superficial FDC networks. White arrowheads point to a large blood vessel, used as a landmark for reorientation over time. Green arrows indicate the borders of AIW FOV. Scale bar, 500 μm. Image was processed with background subtraction, median filtering (Despeckle), linear brightness, and contrast adjustment. See also Figures S1 and S2.

To allow the imaging of the spleen, we developed a tailored surgical protocol based on that of the kidney^41^ (Figures 1A and 1B). We employed a modified titanium window,^42^ retailored from the original AIW,^10^ to enable serial intravital imaging of abdominal organs using an upright two-photon microscope.

### High-temperature PMOXA coating reduces immunogenicity

Implants commonly elicit a foreign-body response involving acute and chronic inflammation, granulation tissue,^43^ and, over time, encapsulation by connective tissue.^11^ This impedes imaging and impacts the systemic immune response. To prevent granulation tissue, we sought to passivate the AIW using a coating consisting of oxidation-stable antifouling brushes of 4.25 kDa poly-2-methyl-2-oxazoline (PMOXA),^44^^,^^45^ with predefined spacing, grafted onto a polyacrylamide (PAcrAm) backbone. High-density PMOXA brushes were grafted to the coverslip surface using high-affinity electrostatic amine linkers and covalent silane linkers designed on the PAcrAm backbone for long-term stability.^46^ Macrophages are key early responders, whereas fibroblasts contribute later with extracellular matrix deposition during the encapsulation process.^43^^,^^47^ We evaluated cellular overgrowth of murine 3T3 fibroblasts and RAW 264.7 macrophages on non-coated glass, room-temperature, and high-temperature (80 °C) PMOXA coating. The high-temperature (80 °C) PMOXA passivation resulted in fewer adherent cells for both cell lines during two weeks of culture (Figures S1A and S1B). Cells exhibited reduced spreading on the passivated surfaces, providing a clearer field of view (FOV) (Figure S1C). High-temperature PMOXA (80 °C) passivation was chosen for subsequent experiments.

### The AIW allows tracking FDC network dynamics over 2 weeks

Mouse models of and patients with SLE commonly display splenic GCs and splenomegaly. The dramatic remodeling of the spleen occurring in connection with an autoimmune response at the same time presents a good proof-of-principle and a challenging test case due to the expansion and contraction of the tissue. To test the feasibility of longitudinal imaging at cellular resolution, we chose *FoxP3*^DTR^^−^^GFP^ reporters marking all T regulatory (T_REG_) cells, including T follicular regulatory (T_FR_) cells in GCs. Mice were treated by the epicutaneous application of the small-molecule TLR7 agonist resiquimod (R848) to commence an SLE-like autoimmune response.^48^ In one cohort, an AIW was implanted over the spleen on Day 4. Another cohort was treated with R848 for 4 weeks, an AIW was implanted on day 26, then treatment was stopped (day 29) (Figure 1C). Mice with an AIW were able to move and climb freely (Figure S2A) and did not show signs of distress or discomfort, and only displayed a transient mild weight loss upon surgery and first imaging sessions (Figures S2B and S2C). Mice undergoing R848 treatment and surgery without AIW implantation displayed no significant weight loss (Figure S2D).

The dynamics of FDC networks were followed *in vivo* for 2 weeks (Figure 1C). GCs were identified based on the presence of autofluorescent vacuolar TBMs^18^^,^^49^ and intravital labeling of the FDC network with anti-CD35/complement receptor 1 (CR1)-iFluor647 (Figure 1C). PT-tracks and the PALS were identified by second-harmonic generation (SHG) and dense GFP^+^ populations, respectively. Individual GCs had to be reidentified every imaging day despite significant tissue changes. Therefore, a 250-μm-thick 3D overview was acquired at the beginning of each imaging session (Figure 1D). In the first imaging session, the most superficial GCs were selected. In the following imaging sessions, these were reidentified in the 3D overview based on landmarks such as vasculature, other GCs, the capsule, or the borders of the AIW FOV (Figure 1D). Collagen trabeculae, visualized by SHG, were used to reposition the GC into the same imaging position by aligning the collagen fibers in relation to the border of the FOV.

### Follicles and FDC networks remodel extensively

**Figure 2:**
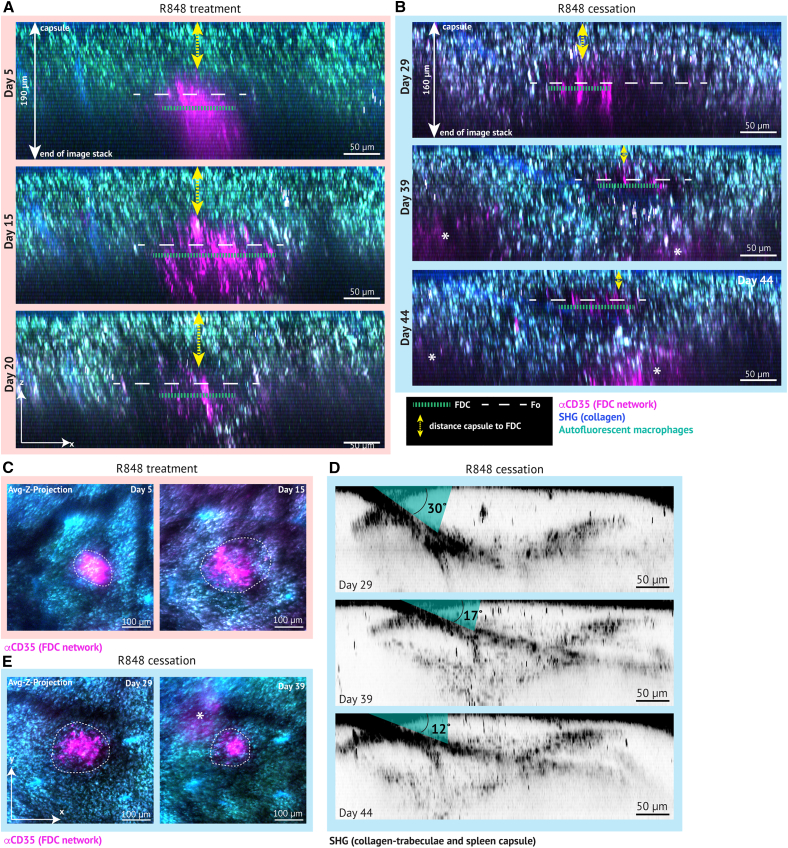
Extensive remodeling of FDC networks and follicles (A) Orthogonal view of autoreactive GCs across 190 μm depth over 2 weeks (d 5–20). Green dashed line indicates the diameter of the FDC network. White dashed line indicates the estimated diameter of the follicle (Fo). Yellow double-arrow indicates the changes in distance from the capsule to the top of the FDC network. Micrographs display orthogonal maximum y-projection of all imaging frames with CD35 signal (see also Figure S3A). Scale bars, 50 μm. (B) As A, but after treatment cessation (day 29–44) across 160 μm depth at all days. Note that deeper GCs rise from below (∗) on days 39 and 44 as the spleen parenchyma contracts. (C) Average z-projection of all imaging frames with CD35 signal on day 5 and day 15 (see also Figure S3B). White dashed circle indicates the labeled area of the FDC network. Scale bars, 100 μm. (D) As C, but after treatment cessation. Note the rise of a deeper FDC network (∗) on day 39. (E) Changes in the splenic collagen trabeculae after treatment cessation. Micrographs are complementary to micrographs in B, pre-processed and displayed similarly, but SHG was isolated and inverted to visualize the capsule and trabeculae. Scale bars, 50 μm. Linear brightness and contrast adjustment. See also Figure S3.

The FDC network and the follicle expanded during R848 treatment (Figure 2A) and contracted after R848 treatment cessation (Figure 2B). This was also evident in an axial x-y-view (Figures 2C and 2D). However, during R848 treatment, non-labeled, dark cells accumulated above the FDC network (Figure 2A, day 20). This pushed the FDC networks deeper into the tissue as the R848 treatment progressed, impeding reliable 3D imaging readouts after day 17. Because areas of interest visualized on previous days moved into non-imageable depths, day 20 was excluded from further image analyses. Nonetheless, our observations indicated that the entire spleen parenchyma, including the red pulp, adapted to the changes in follicle size. To investigate this, we tracked changes in the arrangement of collagen trabeculae in the red pulp surrounding follicles (Figure 2E). The angle of the trabeculae emerging from the capsule decreased over time as the GC contracted, indicating that collagen trabeculae are intimately associated with follicular structures. Taken together, using the AIW with a combination of different intravital labeling techniques and analyses revealed unique longitudinal information of immunological processes in the spleen, including remodeling of GCs and the spleen parenchyma.

### The splenic imaging window enables serial characterization of dynamic cell motility behavior

The true advantage of a splenic AIW lies in the possibility of tracking week-long splenic tissue changes. This can entail, as shown in Figure 2, the capture of changes in follicle and GC size and thus the measurement of immune activity. We demonstrated that the AIW can also be utilized to reliably track cell motility patterns serially over days to weeks. We used both CD8a-cre;tdTom^flx/flx^ mice and adoptively transferred CFSE-labeled B cells to visualize different cell motility patterns in the spleen, especially in splenic follicles serially over time (*n* = 4 mice).

To first assess imaging stability, we performed a 3D 25-minute-long time-lapse movie of the same spleen region. We observed minor z-drift (ca. 10 μm) and some subtle in-plane movement (x/y), primarily caused by respiratory and vascular motion (Video S1, left video). After digital 3D drift correction, the same FOV remained highly stable, enabling accurate longitudinal tracking of individual cell movements (Video S1, right video).

We then assessed whether we could track adoptively transferred CFSE-labeled B cells in the same follicle on different days, despite their deeper localization than the red pulp visualized in Video S1. For this, 200,000 CFSE-labeled B cells were adoptively transferred into CD8a-cre;tdTom^flx/flx^ mice, and timelapses of the same follicle were acquired 1, 2, and 9 days post-adoptive transfer (Video S2). These results confirmed that the AIW setup allows reproducible, stable time-lapse imaging of the same follicle over extended time periods and across multiple imaging sessions.

Free-run single-plane time-lapse imaging visualizing splenic vasculature labeled by 70 kDa FITC-Dextran revealed individual lymphocytes, including CD8 T cells, circumnavigating blood vessels or being carried by the blood flow (Video S3). This demonstrated that the splenic AIW setup can be used for velocity studies in splenic vasculature over multiple days as well.

### T_FR_ cells with a membrane-associated fluorescent protein can be visualized over time in deep spleen tissue *in vivo*

**Figure 3:**
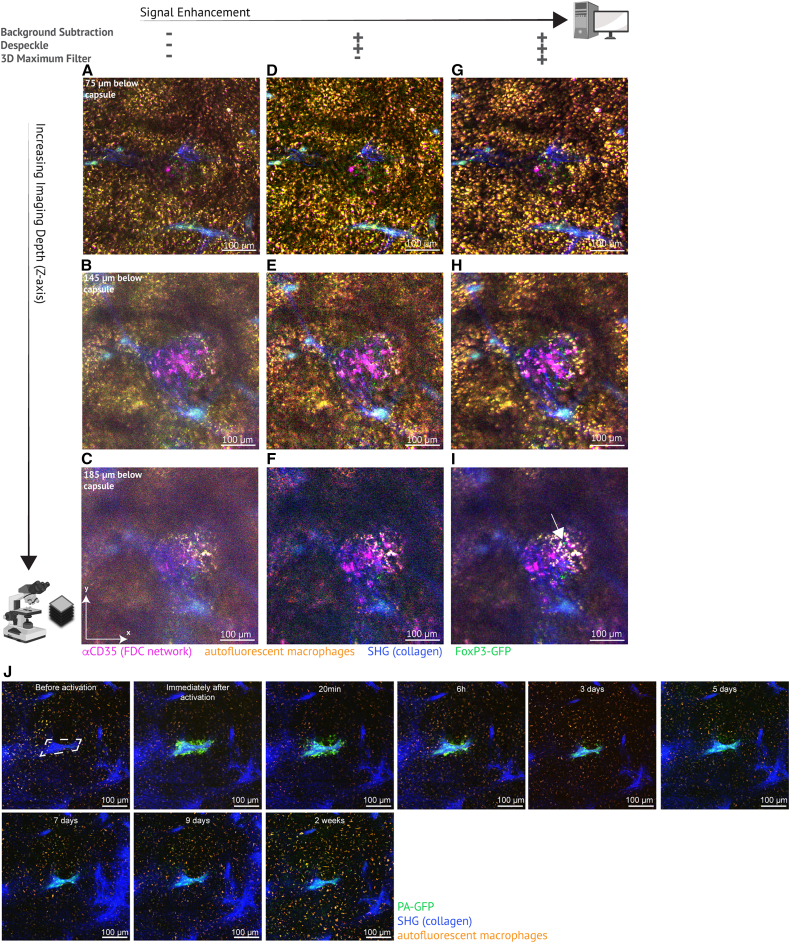
Processing of deep 3D data to enhance single-cell resolution, and spatiotemporal tracking using PA-GFP (A–C) Imaging frames of the same FOV 75, 145, and 185 μm below the capsule (dual track overlay from λ_Ex_ 840 and λ_Ex_ 940). FoxP3-GFP^+^ cells are mostly visible, but with a low signal-to-noise ratio (SNR). Scale bars, 100 μm. (D–F) Same images as A-C, after background subtraction and median filtering (Despeckle). (G–I) Same images as D-F, after 3D Maximum filter. White arrow identifies a FoxP3^+^ cell. Scale bars, 100 μm, calculated by pixels as precise scaling is not possible after 3D maxima filtering due to the spatial enlargement of single spots. (J) Longitudinal tracking of PA-GFP-stimulated area (collagen trabecula) for 2 weeks. Scale bars, 100 μm. Experimental *n* = 3. All micrographs were adjusted in brightness and contrast (linear); any further processing is indicated in panel legends above.

We used *Foxp3*^DTR−GFP^ fluorescent transgenic reporter mice to visualize T_REG_ cells, including those localizing to GCs, anatomically marking them T_FR_ cells^50^ (Figures 3A–3I). Two-photon excitation enables deep tissue imaging.^51^^,^^52^ However, single-cell resolution is limited due to light scattering in deeper tissue.^52^^,^^53^ GCs mostly occur deeper than 100 μm below the capsule. T_FR_ cells localize equally deep, and T_FR_ cells of *Foxp3*^DTR−GFP^ mice emit smaller spots of dimmer signal than, e.g., the CD8 T cells in CD8a-cre;tdTom^flx/flx^ mice because their GFP expression is dimmer and confined to the cell membrane only. To visualize T_FR_ cells of *Foxp3*^DTR−GFP^ mice reliably at all imaging depths, we developed an image processing protocol to enhance contrast and signal-to-noise ratio (SNR) and increase both size and signal of small, dim features. The effect of image processing on dim membrane-associated GFP-emission of both the T_FR_ cells and the labeled FDC network can be seen in Figures 3A–3I at three different tissue depths. This demonstrated the feasibility of achieving single-cell resolution at around 200 μm depth of dim and spatially restricted fluorescent proteins using the AIW.

### Photoactivation enables precise spatiotemporal tracking of cells over two weeks

Photoactivatable reporters such as PA-GFP have been used extensively for short-term imaging of dynamic cell subsets in the GC or to link anatomical location with *ex vivo* analyses.^28^^,^^54^^,^^55^^,^^56^ The half-life of photoactivated PA-GFP in GC B cells was estimated to be only 30 h,^54^ however, *in vitro* the photoactivated absorbance spectrum was maintained for >1 week at 37 °C.^57^ We tested the longevity of photoactivated PA-GFP using the AIW setup and found that it persists for at least two weeks in sessile, non-dividing cells, such as trabecular fibroblasts (Figure 3J), capsular fibroblasts, or quiescent endothelium (not shown). Hence, leveraging the AIW, it is possible to monitor such spatially restricted subsets of cells over weeks *in vivo*.

### FDC network expansion and contraction are reflected macroscopically

**Figure 4:**
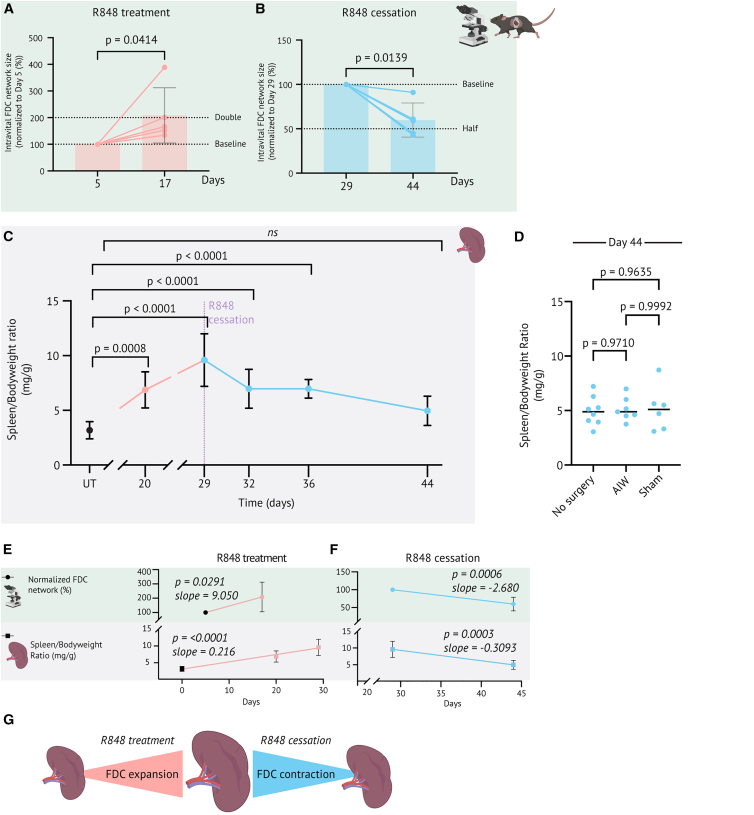
FDC network expansion and contraction are reflected macroscopically (A) Normalized FDC area based on average z-projections on day 5 and 17 during R848 treatment (*n* = 6, 2 cohorts with 3 mice each; 1 exclusion for technical reasons on day 17). (B) As A, but during R848 cessation, day 29 and 44 (*n* = 6, 2 cohorts with 3 mice each; day 17, 1 exclusion for technical reasons). Data in A and B are normalized to the baseline measurement of individual mice on days 5 and 29, respectively. Mean and SD are indicated for each group. *P*-values were computed on non-normalized, raw measured area values (Figure S4) with a two-tailed, paired *t*-test. (C) Spleen to bodyweight ratios of untreated (UT) mice, mice during R848 treatment, and after R848 cessation. Day 20 was measured on AIW mice; all other time points were from non-surgery mice. Mean ± SD from 4 to 11 mice from 2 to 4 cohorts per timepoint. *P*-values were computed with ordinary one-way ANOVA and Dunnett’s post-test, comparing all time points to UT. (D) Comparison of spleen/bodyweight ratios of non-surgery, AIW-implanted, and sham mice after R848 cessation, day 44. Data pooled from 2 to 5 cohorts with n = 6–8 mice per group. (E) Comparison of FDC network (green background) and spleen/bodyweight ratio (gray background) changes during R848 treatment. Slopes were calculated with simple linear regression analysis of each dataset, and *p*-values represent the significant deviation from zero. (F) As E, but after R848 cessation. (G) Graphical summary of the correlation between changes in the FDC network and spleen size. See also Figure S4.

We next examined whether the changes in the FDC network correlated with macroscopic changes in spleen weight in matched cohort studies. The FDC network was outlined and area-quantified on average z-projections (Figures S3, 2C, and 2D). This revealed that it doubled from day 5 to day 17 (Figures 4A and S4A) and halved from day 29 to day 44 (Figures 4B and S4B).

Splenomegaly is a hallmark of SLE,^58^ known to be triggered by R848 treatment.^48^^,^^59^ Significant splenomegaly was observed 20 and 29 days into R848 treatment, then gradually reverted after the cessation of R848 treatment on day 29, reaching non-significance compared to baseline on day 44 (Figure 4C). Importantly, spleen size on day 44 was comparable between AIW-implanted, sham-operated, and non-surgery mice (Figure 4D). Together, this confirmed that both expansion and contraction of the spleen could occur in the presence of an AIW implant. Moreover, the FDC network expansion correlated with the spleen expansion, whereas its contraction correlated with the reversion of splenomegaly (Figures 4E–4G).

### FDC network expansion and contraction mirror GC cell dynamics

**Figure 5:**
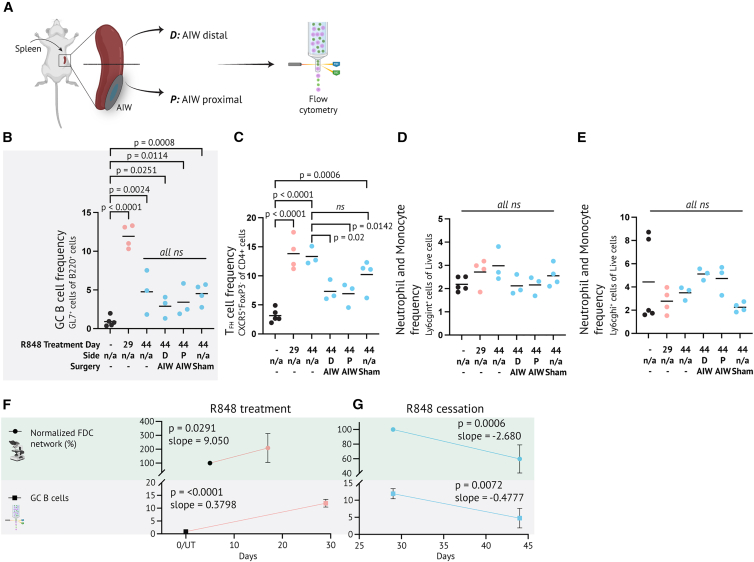
FDC network expansion and contraction mirror GC cell dynamics (A) Schematic overview of sampling for flow cytometry. Spleens from AIW mice were divided into an AIW-proximal (attached to AIW) and a distal part. (B) Flow cytometry analysis of GC B-cell frequencies in the spleen. Individual datapoints and mean from 3 to 5 mice from 1 to 2 cohorts, per group. (C) As B, but for T_FH_ cells. (D) As B, but for Ly6gc^int^ cells. (E) As B, but for Ly6gc^hi^ cells. In B-E, *p*-values were computed with One-way ANOVA comparing to untreated controls with Dunnett’s post-test. Furthermore, surgery and non-surgery mice were compared at day 44. (F) Comparison of FDC network and GC B-cell frequency changes during R848 treatment. (G) As F, but for R848 cessation. Slope values were calculated with simple linear regression analysis of each dataset, and *p*-values represent the significant deviation from zero. See also Figure S5.

To determine if the FDC network AIW imaging read-outs were robust and reflected GC cell expansion, we performed flow cytometry analyses on day 29 and 44 AIW mice, including matched untreated controls and day 44 sham- and non-surgery mice. The spleen of AIW mice was divided into the part attached to the AIW and the part distal from the AIW, to investigate if the window attachment influenced the underlying tissue (Figure 5A). The flow cytometry analyses (gating strategy in Figure S5) showed an elevated GC B-cell level on day 29, which had decreased to approximately the same level on day 44 across sham-, non-surgery, and AIW groups, yet remained above that of untreated mice (Figure 5B). T_FH_-cell frequencies also increased during R848 treatment and slightly decreased after R848 treatment cessation. The decrease in T_FH_ cells in AIW mice (both window-proximal and distal spleen parts) seemed slightly larger than in sham- and non-surgery mice (Figure 5C). There was no major inflammatory response associated with the AIW implant itself, neither distally nor proximally (Figures 5D and 5E). Whereas Ly6cg^hi^ neutrophil and monocyte frequencies were similar, AIW mice had slightly higher Ly6cg^int^ frequencies than sham- and non-surgery mice at day 44, but compared to untreated controls, there was no significant difference (Figures 5D and 5E). The changes in GC B-cell frequencies correlated with those in the FDC network; both increased during R848 treatment and decreased following R848 cessation (Figures 5F and 5G). Taken together, this verified that the AIW did not perturb the immune response and validated the immunophenotype readout.

### FDC network dynamics track with dark zone expansion and contraction

**Figure 6:**
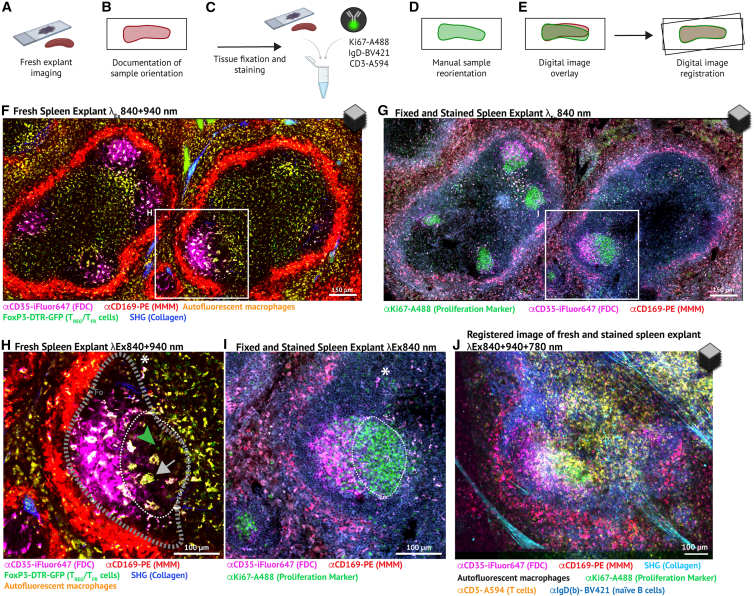
FDC network dynamics track with dark zone expansion and contraction (A–E) Schematic overview of experimental setup. (F) Overview of two white pulp areas of a fresh spleen explant, showing several FDC networks (CD35^+^). MMM, marginal zone metallophillic macrophages; SHG, second harmonic generation. Scale bar, 150 μm. (G) Same area overview as F, after tissue fixation and staining with Ki67-A488 to visualize proliferating cells. Scale bar, 150 μm. (H and I) Cut-out and magnification of the same areas of F and G, respectively. Dark zone outlined by a dotted line in white, and additional CD35-positive and -negative areas of the follicle in gray. The white asterisk points to the same macrophage, indicating the same focal plane is in focus in the fresh (H) and fixed (I) spleen section. Scale bars, 100 μm. In H, the gray arrow points to a TBM, and the green arrowhead to a T_FR_ cell. (J) Overlay of fresh and fixed micrograph with registration. Note the rotation of different imaging tracks along the bottom edge of the image. All micrographs in this figure visualize a single focal plane out of a 100 μm 3D z-stack from 3 to 4×2 tiles. Stitched micrographs were median filtered (Despeckle) and adjusted in brightness and contrast (linear). See also Figure S8.

A dark region surrounding the labeled FDC network expanded during R848 treatment and contracted following the cessation of treatment (Figures 2A and 2B). We hypothesized that this dark area encompassed both the mantle zone and the GC dark zone. To test this hypothesis, we used thick explants to increase the 3D multiplexing potential^60^ (Figures 6A–6I). Prior to euthanasia, αCD169-PE was administered to label marginal zone metallophilic macrophages (MMMs) in addition to the FDC-labeling with αCD35-iFluor647 (Figures 6F and 6H). After imaging freshly explanted spleen sections, the tissues were fixed and stained with the proliferation marker αKi67-A488 (Figures 6A–6C, 6G, and 6I). Spleen slices were positioned to ensure accurate overlay of sequentially acquired images, followed by digital image registration (Figures 6D and 6E). This identified dark regions around the FDC network and TBMs (Figures 2A, 2B, 6F, and 6H) with the dark zone of GCs (Figures 6G and 6I). Staining with αCD3 and αIgD confirmed that residual dark areas were part of the follicular region (IgD^+^, Figure 6J) rather than the PALS (CD3^+^, Figure 6J).

### The systemic immune environment is not perturbed

**Figure 7:**
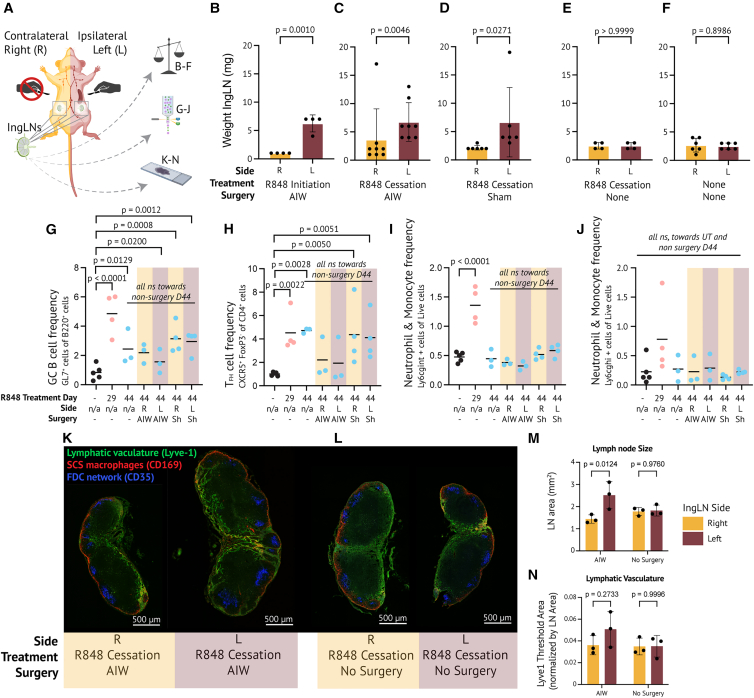
The AIW does not initiate a systemic immune response (A) Schematic overview of experiment: skin-draining inguinal lymph nodes (IngLNs) from both the ipsilateral surgery side (left - L) and the contralateral non-surgery side (right - R) were harvested on the last day of imaging experiment (2–2.5 weeks after surgery and 6 imaging sessions per mouse, day 20 and day 44). (B) IngLN weight of AIW R848 treatment group (*n* = 4 from 2 cohorts). (C) IngLN weight of AIW R848 cessation group (*n* = 8 from 5 cohorts). (D) IngLN weight of sham-surgery R848 cessation group (*n* = 6 from 3 cohorts). (E) IngLN weight of non-surgery R848 cessation group (*n* = 4 from 3 cohorts). (F) IngLN weight of non-surgery untreated group (*n* = 6 from 2 cohorts). In B–F, mean ± SD, statistical analysis performed by two-tailed paired *t*-test on log-transformed data. (G) GC B-cell frequencies in IngLNs. (H) T_FH_ frequencies in IngLNs. (I) Ly6gc^int^ frequencies in IngLNs. (J) Ly6gc^hi^ frequencies in IngLNs. Data in G-J were from 3 to 5 mice per group (1–2 cohorts). Individual datapoints are represented with the mean, and *p*-values were computed with one-way ANOVA with Dunnett’s post-test comparing to untreated controls. Furthermore, the surgery mice were compared to the non-surgery mice on day 44 as well. Only significant *p-*values shown. (K) Representative micrographs of right and left IngLN from an AIW mouse at day 44, stained for CD35 (blue, FDC network), CD169 (red, subcapsular sinus (SCS) macrophages), and Lyve-1 (green, lymphatic vasculature). Scale bars, 500 μm. Micrographs are stitched confocal tile scans, adjusted in brightness and contrast. (L) As K, but for a non-surgery mouse. (M) IngLN area by histology. (N) Lyve1^+^ area normalized to the LN area. In M and N, *n* = 3 mice per group, all IngLN were harvested from the R848 treatment cessation regimen groups on day 44. Mean ± SD, with *p*-values determined using two-way ANOVA and Šídák’s multiple comparisons test comparing right and left IngLN in each group. All *p*-values shown.

To examine a potential immunological impact of the AIW, we compared the skin-draining inguinal lymph nodes (IngLNs) on the surgery side (ipsilateral, left) and non-surgery side (contralateral, right) (Figure 7A). We furthermore investigated the influence of surgery in general and of the AIW specifically by comparing the IngLNs of AIW, sham, and non-surgery control mice. Two weeks after AIW implantation (day 20 R848 treatment, day 44 R848 cessation) or sham-surgery, the left IngLN was significantly heavier in all three surgery groups; however, comparable between AIW and sham-surgery (Figures 7B–7D). No side differences were seen in untreated and R848-treated non-surgery mice matched on day 44 (Figures 7E and 7F).

R848 treatment induced a robust GC B-cell and T_FH_ cell response in IngLN, which reverted upon R848 cessation, albeit not fully to baseline levels by day 44 (Figures 7G and 7H). GC B-cell and T_FH_ cell levels in sham-, non-surgery, and AIW groups were not different on day 44 (Figures 7G and 7H), moreover, there were no significant differences in neutrophil frequencies between these groups at day 44 (Figures 7I and 7J). However, there was a transient influx of neutrophils at the peak of autoimmune inflammation on day 29, which reverted to control levels on day 44 (Figures 7I and 7J). Flow gating strategies can be found in Figure S5. Confocal immunofluorescence microscopy of the lymphatic vasculature in IngLNs of AIW and non-surgery mice at day 44 showed an increase in Lyve-1 expression in the left AIW node (Figures 7K and 7L). Generally, the left IngLN of AIW mice was larger (Figure 7M), and although the Lyve-1 positive area was increased, this was not significant when normalized for IngLN area (Figure 7N). Overall, these results indicate that the impact of surgical intervention is confined to the ipsilateral side. Immune cell phenotyping showed no differences between surgery and non-surgery mice, and negligible side differences, demonstrating the absence of systemic immune perturbation.

## Discussion

Previous intravital microscopy studies have uncovered several dynamic processes in the spleen, including the entry of T cells via perivascular tracks,^61^ the shuttling of B cells between compartments,^3^ and a mechanosensing mechanism for B-cell retention in the marginal zone triggered by red blood cells.^62^ However, the terminal experimental setups employed in those studies render them less well suited to interrogate the slower dynamics of longer-lasting changes. Ritsma and colleagues developed an AIW that allowed tracking the migration of adoptively transferred B and CD8^+^ T cells into the spleen. They then followed the acute CD8^+^ T-cell response to ovalbumen peptide *in vivo* and were able to quantify a T-cell response 7 days after antigen challenge.^11^ Here we more than double the previous imaging time limit, by demonstrating that serial intravital two-photon microscopy using an AIW allows insight into week-long biological processes, exemplified by the autoreactive FDC network and GC dynamics (Figures 1, 2, 3, and 4), and associated deep tissue changes (Figures 2, 4, and 6). This allows high-dimensional temporospatial insights into important biological processes, such as the break-of-tolerance occurring in autoimmunity, vascular changes in disease, and hematological malignancies affecting the spleen. We also demonstrate the power of our setup in terms of capturing dynamic cell migration processes and do so at different time points within the same animal in the same microanatomical location (Videos S1 and S2).

Whereas titanium, constituting the frame of the AIW, is biocompatible,^63^ the glass coverslip is affected by cellular overgrowth, which can lead to reduced imaging quality, angiogenesis, and an untoward immune response. This necessitated the implementation of an anti-fouling method to dampen the immunostimulatory activity of the window.^47^ We found that a high-temperature PMOXA passivation alleviates untoward immune reactions (Figure S1), additionally prolonging the imaging period after implantation by preventing organ encapsulation. The immunostimulatory effect of R848 treatment was far stronger than any impact of the AIW itself, as measured by, e.g., the GC B-cell response. This aligned well with our phenotyping of the immune environment in the spleen, which showed that the implant and the repeated intravital imaging did not alter general immune responses nor trigger a systemic immune response (Figures 5 and 7). Specifically, we compared contra- and ipsilateral skin-draining lymph nodes of the surgical area (Figure 7), and spleen parts attached to the coverslip versus distal to the AIW (Figure 5). Confinement of the inflammation to the ipsilateral (left) skin-draining IngLN was further proof that the AIW does not majorly perturb the immune system and that readouts are not skewed by the implant.

Following surgery, mice exhibited a slight and transient drop in bodyweight (Figures S2B and S2C). Although the first imaging session seemed to induce a slight weight drop, potentially as mice had to adapt to the procedure initially, the repeated 2-h anesthesia during subsequent intravital imaging sessions did not affect the body weight. The ability of mice to thrive on this protocol is not only desirable from an ethical perspective but is also envisioned to deliver more reproducible results.^64^^,^^65^ Transient leukocyte trafficking into a surgical site decreases 72 h post-surgery and is significantly increased by stress.^66^ Accordingly, in the R848 cessation cohort, operated mice were given a 72 h rest before intravital imaging was commenced. However, due to technical challenges associated with the expansion of the spleen over time, this was not possible for the R848 initiation cohort, which was given only a single day of rest. Due to the affixture of the spleen to the AIW, the parenchyma cannot expand laterally, only depth-wise, causing a faster decline in the imaging quality in depth upon R848 treatment initiation (Figure 2A). In the cessation cohort, the initial attachment of the spleen to the AIW was performed on the already expanded spleen, preempting this issue. To capitalize on imaging readouts as long as possible, it was necessary to implement a shorter rest period after surgery in the R848 treatment cohort.

As the red pulp in the superficial areas of the spleen is extremely uniform without any specific landmarks, identifying the same GC again and again over several weeks is challenging. A number of techniques have been described in the field of serial intravital imaging to reidentify previously imaged areas, such as numbered gridded coverslips,^10^^,^^67^ microcartography based on deposited microspheres,^68^ or laser-injury marks.^41^ We specifically refrained from the latter two as implanted microspheres and laser-induced injuries could have perturbed the immune response. Creating a 3D tiled overview map at the beginning of every imaging session, capturing deep labeled FDC networks in the window area, was a suitable strategy for reidentifying GCs in the spleen (Figure 1D). The overview map served to pinpoint the position of each GC in relation to the window position. The stable location of GCs relative to the architecture of rare, large blood vessels further facilitated their reidentification. This approach reliably identified the same GCs over time, except when a FOV rotated out of sight, or the GC of interest was pushed too deep into the parenchyma due to heavy spleen remodeling, as described above.

Through FDC labeling, we could identify the light zone of GCs (Figures 1 and 2) and further the entire follicle, including the GC, based on the lack of red pulp macrophages in the dark area adjacent to the FDC network (Figures 6F–6I). We were hence able to capture both autoreactive GC expansion (Figures 2A and 2C) and autoreactive GC contraction (Figures 2B–2D–2E). Single cells of interest could also be detected in the GCs (Figures 3A–3I). Despite being attached to the AIW, the spleen can remodel heavily, stay intact, and does not rupture while remodeling (Figures 4C and 4D). In fact, the spleen could both expand and contract with an AIW attached, as the spleen/bodyweight ratio doubled from baseline (untreated) to day 20 (end of R848 initiation period) and contracted to half its maximal size in just 2 weeks after R848 cessation (day 29 to day 44) (Figure 4C). However, the extensive tissue remodeling, especially during the expansion of GCs, caused a slow decrease in imaging quality over time as more tissue, potentially proliferating B cells, accumulated above the area of interest (Figure 2A, day 20).

By comparing spleen metrics and flow cytometry results to serial intravital images, we confirmed that GC dynamics observed longitudinally intravitally (Figures 2 and 4) correlated with developing splenomegaly and its reversion (Figure 4) and to changes in flow typing results, such as GC B-cell frequencies of matched cohort studies (Figure 5). These comparisons underline that the initiation and contraction of an autoreactive immune response can be captured intravitally. We furthermore showed that intravitally detected changes in the light zone and the darker areas nearby can be indirectly linked to changes in the dark zone of GCs, as we associated the dark areas with the dark zone of GCs (Ki67^+^) of spleen explants (Figure 6). This demonstrates that longitudinal changes of the light zone mirror changes of the dark zone, making intravitally detected changes in FDC networks a correlate of changes in proliferating GC B-cell frequencies (Figures 5 and 6). In fact, FDCs actively shape and support GC reactions through the presentation of intact antigen and the secretion of chemokines and cytokines that attract, retain, and sustain GC B cells.^69^^,^^70^ FDC-derived cytokines, such as interleukin-6 (IL-6) or BAFF, promote B-cell differentiation, proliferation, and survival,^70^^,^^71^^,^^72^^,^^73^ and FDC expression of adhesion molecules ICAM-1 and VCAM-1 supports the physical interaction with GC B cells.^74^ FDCs have furthermore been reported to produce type I interferons, such as IFN-α, supporting autoreactive GC responses.^75^ These findings raise the possibility that the different degrees of FDC network expansion we observed in Figures 4, 5, and 6 may modulate GC dynamics directly, with larger FDC networks providing stronger cell adhesion, chemokine- and cytokine-mediated support to GC B cells and GC reactions, and smaller FDC networks offering reduced support.

The AIW we implement here is similar to that presented in pioneering work by Ritsma et al. 2012 and 2013,^10^^,^^11^ the main difference being the inclusion of “wings” that enable sliding insertion into a 3D-printed holder, allowing stable imaging on an upright microscope.^42^ Upright microscopes display a greater degree of flexibility compared to inverted microscopes, improving the general accessibility of our method. The surgical protocol is similar, relying on a purse-string suture to secure the AIW. However, we also use a non-removable glass coverslip and fix the tissue to the rim of the AIW and the edge of the coverslip, as opposed to the use of a removable glass coverslip, which limits tissue fixation. To obviate the need to periodically replace the coverslip, we use a PMOXA coating with much increased stability compared to PEG. In terms of our results, we demonstrate repeated imaging of the same microanatomical location in the spleen, both by static and dynamic imaging, over a period of two weeks, significantly surpassing what was previously reported.^10^^,^^11^ Notably, we show the stability of our setup specifically under conditions where the spleen is remodeling extensively and expanding dramatically, something which could not be done before. Finally, we provide a much more comprehensive characterization of the local and systemic immune response in AIW mice and sham-operated controls, demonstrating that our AIW protocol does not perturb immune homeostasis.

While we will refrain from outlining technical challenges that most intravital imaging experiments have in common,^76^^,^^77^ there are potential biological limitations that are specific to this method. Even though we significantly extended the imaging period to catch dynamic intravital remodeling, 2 weeks is still a narrow window in perspective of the general development of e.g., autoimmune diseases, in which autoantibodies often gradually increase for months in mouse models and years in asymptomatic patients leading up to clinical diagnosis.^78^ Moreover, despite many similarities between the immune response in mouse and man,^79^ there are also many significant differences.^80^ Perhaps more importantly, the microanatomy of the spleen differs, mainly in the spatial arrangement of B- and T-cell zones.^81^^,^^82^ This notwithstanding, the functional spleen microanatomy is still poorly defined,^81^ and methodology such as the approach presented here might improve functional anatomical understanding.

In conclusion, serial intravital imaging of the spleen is a tool in the investigative toolbox of immunologists that may help shed light on long-lived and dynamic immune reactions in the spleen without perturbing the immune system. It may find use in other areas as well, such as hematology, oncology, and infectious disease research.

### Limitations of the study

This study has biological and methodological limitations. Although we extended the imaging period to two weeks to capture dynamic intravital remodeling, this remains a relatively short window compared to the gradual development of chronic autoimmune diseases, which can evolve over months in mice and years in humans. Our approach, therefore, primarily captures early remodeling events rather than long-term pathological progression. In addition, while murine and human immune systems share core principles, important interspecies differences exist, including distinctions in splenic microanatomy and the spatial organization of B- and T-cell zones. These differences may limit the direct translation of our findings. Finally, to assess the potential perturbation of the spleen induced by the AIW implant, we relied solely on flow cytometric analyses and did not perform complementary histological evaluation. Thus, subtle tissue alterations cannot be formally excluded.

## Resource availability

### Lead contact

Requests for further information and resources should be directed to and will be fulfilled by the lead contact, Søren E. Degn (sdegn@biomed.au.dk).

### Materials availability

This study did not generate new unique reagents.

### Data and code availability

This paper does not report any standardized datasets. A short custom Python script, which was used to run the nuclei model on Google Colab, is included as Data S1. Any additional information required to reanalyze the data reported in this paper is available from the lead contact upon request.

## Acknowledgments

We thank the Laboratory Animal Facility, the Bioimaging Core Facility, and the FACS Core at Aarhus University for excellent technical assistance and support. We thank the technicians at the Department of Biomedicine for excellent support in custom 3D printing. Several scientific icons used in schematic illustrations were obtained from BioRender.com. Funding for this study was obtained from: 10.13039/501100012331LEO Foundation grant LF-OC-22-000977 (SED); The Independent Research Fund Denmark IRFD grant DFF-FSS: 8124-00001 (SED); The Independent Research Fund Denmark IRFD grant DFF-FSS: 9060-00038 (SED); The Novo Nordisk Foundation grant NNF17OC0028160 (SED); The Novo Nordisk Foundation grant NNF19OC0058454 (SED); EFIS-IL Short Term Fellowship (LP); The Danish National Research Foundation center grant CellPAT (DNRF135) (DuS/SED); 10.13039/501100003554Lundbeck Foundation post-doctoral fellowship grant R303-2018-3415 (CFH); 10.13039/501100009708Novo Nordisk Foundation grant NNF19OC0054899 (IMS).

## Author contributions

Conceptualization, L.P., T.R.W., and S.E.D.; methodology, L.P., T.R.W., A.S., K.S.K., C.F.H., S.A.P., J.K.D., Do.S., I.M.S., and A.P.; investigation, L.P., T.R.W., A.S., K.K., C.F.H., S.A.P., and J.K.D.; visualization, L.P., A.S., and S.E.D.; supervision, Du.S., S.F.G., and S.E.D.; writing – original draft, L.P., A.S., and S.E.D.; writing – review and editing: all authors.

## Declaration of interests

The authors declare no competing interests.

## Declaration of generative AI and AI-assisted technologies in the writing process

During the preparation of this work, the authors made limited use of ChatGPT 5.2 to rephrase, refine, and correct text elements. After using this tool, the authors reviewed and edited the text as needed and take full responsibility for the content of the publication.

## STAR★Methods

### Key resources table

**Table:** 

REAGENT or RESOURCE	SOURCE	IDENTIFIER
**Antibodies**		

BV510 Rat Anti-Mouse B220/CD45R (Flow: 1/500)	BD Biosciences	Cat# 563103; RRID: AB_2738007
Qdot™ 605 Rat Anti-Mouse CD4 Monoclonal Antibody (Flow: 1/500)	Thermo Fisher Scientific	Cat# Q10092; RRID: AB_10374736
PerCP/Cyanine5.5 Rat Anti-mouse CD8a Antibody (Flow: 1/500)	BioLegend	Cat# 100734; RRID: AB_2075238
PE/Cyanine7 Rat Anti-mouse CD38 (Flow: 1/500)	BioLegend	Cat# 102718; RRID: AB_2275531
PE Hamster Anti-Mouse CD95 (Flow: 1/500)	BD Biosciences	Cat# 554258; RRID: AB_395330
BV650 Rat Anti-Mouse CD138 (Flow: 1/500)	BD Biosciences	Cat# 564068; RRID: AB_2738574
Biotin Rat Anti-Mouse CD185 (CXCR5) (Flow: 1/200)	BD Biosciences	Cat# 551960; RRID: AB_394301
BV421 Streptavidin (Flow: 1/500)	BD Biosciences	Cat# 563259; RRID: AB_2869475
BV786 Rat Anti-Mouse CD25 (Flow: 1/500)	BD Biosciences	Cat# 564023; RRID: AB_2738548
APC-R700 Rat Anti-Mouse Ly-6G Ly-6C (Flow: 1/500)	BD Biosciences	Cat# 565510; RRID: AB_2739274
Alexa Fluor® 647 Rat Anti-Mouse T- and B-Cell Activation Antigen (Clone GL7) (Flow: 1/500)	BD Biosciences	Cat# 561529; RRID: AB_10716056
Purified Rat Anti-Mouse CD35	BD Biosciences	Cat# 558768; RRID: AB_397114
PE Rat Anti-mouse CD169 (Siglec-1) Antibody	BioLegend	Cat# 142404; RRID: AB_10915697
Purified Rat Anti-Mouse CD16/CD32 (Mouse BD Fc Block™) (1/50)	BD Biosciences	Cat# 553142; RRID: AB_394656
Ki-67 Monoclonal Antibody (SolA15), Alexa Fluor™ 488	Thermo Fisher Scientific	Cat# 53-5698-82; RRID: AB_2802330
BV421 Mouse Anti-Mouse IgD[b]	BD Biosciences	Cat# 742383; RRID: AB_2740739
Alexa Fluor® 594 Rat Anti-mouse CD3 Antibody	BioLegend	Cat# 100240; RRID: AB_2563427
eFluor™ 450 Rat Anti-Mouse LYVE1 Monoclonal Antibody (ALY7)	Thermo Fisher Scientific	Cat# 48-0443-82; RRID: AB_2784723

**Chemicals, peptides, and recombinant proteins**		

Resiquimod (R848)	Sigma-Aldrich	SML0196-50 MG
Viability Dye eFluor780 (1/2000)	Invitrogen™ eBioscience™	Cat# 65-0865-18
PAcrAm™-g-(PMOXA,NH2,Si)	SuSoS AG - Switzerland	N/A
DAPI	Sigma-Aldrich	D9542
Atto-488 Phalloidin	AttoTec, Germany	N/A
iFluor™ 647 succinimidyl ester	AAT Bioquest	Cat# 1031
ViaStain AOPI Staining Solution	Revvity Health Sciences Inc	Cat# CS2-0106-S
Carboxyfluorescein diacetate succinimidyl ester (CFSE)	BD Horizon	Cat# 565082
Fluorescein isothiocyanate–dextran	Sigma-Aldrich	FD70S-1G

**Critical commercial assays**		

B Cell Isolation Kit, mouse	Miltenyi Biotec	Cat#130-090-862

**Experimental models: Cell lines**		

RAW 264.7 macrophages	Sigma Aldrich 91062702-1VL	Sigma Aldrich 91062702-1VL
NIH 3T3 fibroblasts (ATCC origin)	Kindly donated by Fiona Watts lab	Kindly donated by Fiona Watt lab

**Experimental models: Organisms/strains**		

Mouse: *Foxp3*^DTR-GFP^; B6.129(Cg)-*Foxp3*^tm3(Hbegf/GFP^^)Ayr^/J	The Jackson Laboratory	JAX strain no. 016958
Mouse: PA-GFP; B6.Cg-Ptprc^a^ Tg(UBC-PA-GFP)1Mnz/J	The Jackson Laboratory	JAX strain no. 022486
Mouse: CD8a-Cre; B6.Cg-Tg(Cd8a-cre)1Itan	The Jackson Laboratory	JAX strain no. 008766
Mouse: tdTomato^flx/flx^; Gt(ROSA)26Sor^tm14(CAG-^^tdTomato^^)Hze^/J	The Jackson Laboratory	JAX strain no. 007914

**Software and algorithms**		

Cellpose v3.0	Cellpose	https://www.cellpose.org/
Custom Python script for running Cellpose on Google Colab	Custom	Data S1
Imaris v8.2.1	Imaris	https://imaris.oxinst.com/
Fiji v2.14.0/154f	ImageJ/FIJI	https://imagej.net/software/fiji/
FlowJo v10.8.1	FlowJo	http://www.flowjo.com
GraphPad Prism v. 10.1.1	Graphpad	https://www.graphpad.com/features

**Other**		

Custom-made winged titanium rings	SOLIDPART Denmark	N/A
n-Butyl cyanoacrylate glue (Super Glue Glass)	Loctite®	2954982
12 mm ø wide glass coverslips	Menzel Gläser	vwr^TM^ 631-0713
6-0 non-resorbable monofilament prolene suture	Ethicon	8695G
6-0 resorbable vicryl coated suture	Ethicon	J670G
MACS LS Columns	Miltenyi Biotec	Cat#130-042-401
Brilliant Stain Buffer	Invitrogen	Cat#00-4409-42
Buprenorphine 0.3 mg/mL	Bupaq, VETiSearch	N/A
Meloxicam 5 mg/mL	Metacam, Boehringer Ingelheim Vetmedica GmbH	N/A

### Experimental model and study participant details

#### Animals

*Foxp3*^DTR−GFP^ (B6.129(Cg)-*Foxp3*^*tm3(Hbegf/GFP)Ayr*^/J) breeding pairs were purchased from The Jackson Laboratory (strain no. 016958)^83^ and then bred in-house. *Foxp3*^DTR−GFP^ mice express a knocked-in human diphtheria toxin receptor (DTR) fused to a green fluorescent protein (GFP) in all Foxp3^+^ cells without disrupting normal *Foxp3* expression. This allows visualization of T_REG_ and T_FR_ cells. The knocked-in human DTR receptor furthermore allows a targeted ablation of FoxP3^+^ cells with diphtheria toxin, this approach was however not used in these experiments as the strain was only used to visualize FoxP3^+^ cells. To ensure DTR-GFP expression throughout the entire peripheral T_REG_ cell population, female mice were bred homozygously and male mice hemizygously for the X-linked knock-in allele.

Ubiquitin C (UBC) photoactivatable (PA)-GFP (B6.Cg-*Ptprc*^*a*^ Tg(UBC-PA-GFP)1Mnz/J) breeding pairs were purchased from The Jackson Laboratory (strain no. 022486)^54^ and then bred in-house. UBC PA-GFP mice express photoactivatable GFP under the control of the ubiquitin C promoter. Two-photon stimulation in the λ_Ex_ = 720–840 nm range photoconverts PA-GFP, shifting its absorption maximum and enabling subsequent excitation at λ_Ex_ = 940 nm, allowing rapid, stable fluorescent labeling with microanatomical precision.^54^ CD8a-cre mice (B6.Cg-Tg(Cd8a-cre)1Itan) were purchased from The Jackson Laboratory (strain no. 008766)^84^ and crossed to a tdTomato-reporter line (Gt(ROSA)26Sor^tm14(CAG-tdTomato)Hze^/J), which was kindly provided by Ina Maria Schiessl (JAX strain no. 007914).^85^ Mice were bred in-house to obtain CD8a-cre;tdTom^flx/flx^ mice. CD8a-cre;tdTom^flx/flx^ mice constitutively express the fluorescent protein tdTomato in mature single-positive CD8 T cells and were implemented to investigate cell migration dynamics via time-lapse 2-photon microscopy. Mice were housed under specific-pathogen-free (SPF) conditions in individually ventilated cages under regulated and stable temperature and humidity. Offspring were weaned at 3 weeks of age and mice were kept on a 12-h light/dark cycle with standard chow and water *ad libitum*. Throughout the study, animals were monitored daily for signs of distress or non-thriving behavior, including weight loss exceeding 20%, loss of the abdominal imaging window, and reduced activity or failure to thrive. No animals reached the predefined humane endpoints necessitating early euthanasia. All experimental procedures involving animals in this study were approved by local authorities (Animal Experiments Inspectorate, Denmark, license number: 2022-15-0201-01288). Animals were age- (7–14 weeks at initiation of experiments) and sex-matched. Details of experimental and biological replicates are indicated in respective figure legends.

#### Cell lines

RAW 264.7 macrophages (passages 4–12) were obtained from Sigma Aldrich, NIH 3T3 fibroblasts (passages 11–18) originated from ATCC and were kindly provided by Fiona Watt. Cell lines were not independently validated, nor were they subjected to mycoplasma testing.

### Method details

#### Resiquimod (R848) treatment

Mice were briefly anesthetized in continuous flow of 4% isoflurane in atmospheric air, then treated topically on the right ear with 1 mg R848 (Sigma-Aldrich)/mL acetone. Treatment was performed using a 15 cm, latex- and PVC- free cotton-tipped applicator, which was dipped into the R848 solution until soaked, then rolled on the inner leaflet of the ear. This process was repeated on the outer leaflet of the ear. For intravital observation of the R848 treatment cohort, mice were treated twice before starting the observational period and then treated 3x per week until the end of experiment. For the R848 cessation cohort, mice were treated 3x per week for 4 weeks, then treatment was stopped, and the mice were imaged for two weeks without further R848 treatment.

#### Abdominal imaging window (AIW)

Custom-made winged titanium rings (SOLIDPART Denmark) were adapted^42^ from the original AIW model^10^ to specifically enable serial intravital imaging of abdominal organs using an upright two-photon microscope. The rings are double rimmed with a diameter of 14 mm excluding the wings. A 0.4 mm deep and 12.2 mm ø wide recess is engraved on top of the ring to fit a glass coverslip.^42^ Before each implantation, titanium rings were cleaned with soap and water, sterilized using 70% ethanol, and autoclaved individually. Round 12 mm ø wide glass coverslips were sterilized and passivated with oxidation-stable antifouling brushes to ensure biocompatibility of the glass surface and retaining transparency of the implanted AIW over several weeks. The passivation procedure is described below. Glass coverslips were coated several days before window preparation and stored under clean and sealed conditions at −20°C until use. The day before surgery, the coverslip was glued into the engraved recess of the autoclaved titanium ring under laminar airflow (using approximately 7 μL of n-Butyl cyanoacrylate glue (Loctite Super Glue Glass)), then stored in a sterile-sealed Petri dish at room temperature to allow the development of the glue’s full bond strength overnight.

#### Glass coverslip passivation

Prior to coating, borosilicate glass coverslips (Menzel Gläser, vwr, 631–0713) were cleaned by ultrasonication in acetone and 96% ethanol, followed by drying under N_2_ stream. Each side of the coverslips was plasma cleaned for 15 min using a Diener Femto Plasma Etcher at 100 W at ≈100 mTorr, and an O_2_ flow of 30 SCCM was used to activate the surface for attachment of covalent silane linkers. The plasma-cleaned coverslips were then immediately transferred to a sterile Petri dish in a sterile environment under a laminar airflow bench, and were transferred to a sterile 50 mL Falcon tube containing 0.2 μm filtered solution of 0.1 mg/mL Poly(acryl-amide)-g-(PMOXA, 1,6-hexanediamine, 3-aminopropyldimethylethoxysilane)) PAcrAm-g-PMOXA (NH2,Si), (SuSoS AG - Switzerland) dissolved in 1 mM HEPES buffer at pH 7.4. According to an adapted protocol from Ogaki et al.,^86^ the tubes were incubated for 24 h in a water bath at 80°C to achieve ultra-dense coating and optimize the longevity of the antifouling coating. After 24 h of incubation, the coverslips were carefully removed from the solution, rinsed with an excess of 0.2 μm filtered deionized H_2_O, and dried under N_2_ stream. The room temperature PMOXA coating was prepared similarly but by incubation at room temperature.

#### *In vitro* passivation comparison and analysis

RAW 264.7 macrophages (passages 4–12) and NIH 3T3 fibroblasts (passages 11–18) were used to compare the cellular attachment to the passivated coverslips. The cell cultures were maintained in Dulbecco’s modified Eagle’s medium (high glucose DMEM with GlutaMAX, Thermo Fisher Scientific). Cells were incubated at 37°C with 5% CO_2_ and supplemented with 10% (v/v) fetal bovine serum (FBS), 100 mg/mL penicillin, and 100 mg/mL streptomycin. Cells were passaged by trypsinization when approaching 80% confluence.

#### Three types of glass substrates were prepared for the experiments

(i) high-temperature PMOXA coating, prepared as described in the section glass coverslip passivation;
(ii) room temperature PMOXA coating, prepared identically but incubated with PAcrAm-g-PMOXA (NH_2_,Si) at room temperature; and
(iii) uncoated coverslip controls, which were cleaned by ultrasonication in acetone and ethanol followed by plasma cleaning, but were not incubated in the PMOXA solution.

For *in vitro* experiments, prepared coverslips were mounted into bottomless 96-well plates (Greiner Bio-One, 655000) using laser-cut double-sided adhesive (ARcare 90106NB). The adhesive was patterned to match the well geometry of the plate to form a stable, watertight seal between each well and the glass substrate (see also^87^).

The cell experiments were performed in the same medium, including 10% FBS, which was used for culturing at a seeding density of 3000 cells/cm^2^. Each day, 2/3 of the medium in each well was replaced with fresh medium. On days 4 and 8, an additional 3000 cells/cm^2^ were seeded to ensure the supply of fresh viable cells on the non-adhering surfaces.

After 14 days, the cells were fixed for 10 min in 4% PFA and permeabilized in 0.2% Triton X-100 for 10 min before staining for nuclei with 300 nM DAPI (Sigma-Aldrich) and for F-actin with 50 nM Atto-488 Phalloidin (AttoTec, Germany) for 30 min. The central 50% of each well was imaged using the high-content imaging system ImageXpress Pico (Molecular Devices) with a 20× objective. The cell experiments were repeated independently three times, with each independent repeat having two experimental repeats per condition. Nuclei images were segmented using Cellpose v3.0.^88^ Pretrained nuclei model on a GPU (Tesla T4) enabled on Google Colab notebook using a custom-made Python script (Data S1) to count the number of the adherent cells. Binary thresholding of pixel intensities above the background gray value of F-actin images was used to estimate the percentage of the surface covered by the cells.

#### AIW implantation over the spleen

A surgical protocol was adapted from the AIW implantation over the left kidney.^41^ Surgical instruments were sterilized in a glass bead sterilizer and all surgical surfaces including the heating plate were cleaned using 70% ethanol. Additionally, a sterile, disposable surgical drape was used to cover the heating plate and working space. Mice were anesthetized with isoflurane in medical air with 50% oxygen (induction: 3.5% isoflurane; maintenance 1.2–1.8% isoflurane; flow rate: 0.6–1.2 L/min, Anesthetic Vaporizer UNO300VAP). Mice received analgesia 15 min prior to surgery with Buprenorphine in 0.9% NaCl (0.1 mg/kg bodyweight i.p.) and were placed on the draped and cleaned heating plate. A nose cone was used to maintain anesthesia, and protective eye ointment (Visc-ophtal øjengel 2 mg/g, Orifarm) was used to prevent corneal ulceration. The surgical area on the left flank including surrounding fur was disinfected with chlorhexidine (local hair removal the day prior to surgery). The AIW was placed between the left ribcage and left hind leg, ventral to the spine (Figures 1A and S6). After loss of pain reflexes, a 1 cm long dorsoventral incision in the left flank was made, cutting under vision through skin, fat tissue and muscle layer, respectively (Figures S6A–S6C). Minor bleeding was stopped with a hemostatic dental sponge (Spongostan, Ethicon). Starting dorsally, a purse-string suture was set around the incision using a 6-0 non-resorbable monofilament prolene suture (Ethicon), connecting muscle and skin layer, while avoiding inclusion of fat tissue (Figure S6D). Then the spleen was carefully mobilized from the abdominal cavity by rolling gently with a sterile cotton swab over the perisplenic fat, combined with a soft pull on the perisplenic fat using dull tweezers. The surgical field was regularly irrigated with droplets of sterile 0.9% NaCl to reduce friction during spleen mobilization (Figure S6E). After successful spleen mobilization, skin around the incision was covered in sterile non-woven cotton tissue strips pre-wetted with sterile 0.9% NaCl (Figure S6F). Then 10 μL of n-Butyl cyanoacrylate glue (Loctite Super Glue Glass), was applied in the inner borders of the AIW sink (where glass coverslip meets the titanium ring) (Figure S6G). The AIW was then glued onto the spleen in one smooth movement and held still for 5-7 min to allow polymerization of the glue (Figure S6H). Finally, the skin was carefully inserted into the grove between the two rims of the implant, and the purse-string suture was tightened and secured with surgical knots (Figures S6I and S6J).

#### Sham surgery

Sham-operated mice were handled similarly to AIW operated mice. After the incision, the spleen was immediately mobilized and exposed similarly to AIW mice without laying a purse string suture beforehand. After the spleen was guided back into the abdominal cavity, muscle and skin layer were closed after another with approximately 10 single stitches per layer. A 6-0 resorbable vicryl coated suture (Ethicon) was used for the muscle layer and a 6-0 non-resorbable monofilament prolene suture (Ethicon) for the skin layer.

#### Pre- and postoperative management

To acclimatize mice to the taste of analgesic drinking water, important to avoid dehydration immediately after surgery, normal drinking water was exchanged with buprenorphine (0.009 mg/mL) drinking water already two days before surgery. Chow was additionally soaked with buprenorphine drinking water to enhance adaptation to taste. One day before surgery, the hair in surgical field (ca. 2 cm × 2 cm) on the left flank was removed under anesthesia. First with an electrical shaver, then with depilation cream. A thin layer of moisturizing skin cream was applied to soothe the skin after hair removal.

Immediately after surgery, mice received meloxicam (1 mg/kg bodyweight s.c.) for additional pain-relief and anti-inflammation. Postoperative analgesia (0.009 mg/mL buprenorphine drinking water and 1 mg/kg bodyweight meloxicam s.c./day) was implemented for 2 additional days after the surgery day. After surgery, mice were placed in a fresh and clean cage under an infrared heating lamp for 30–60 min recovery. Mice were single-housed and monitored daily for appropriate wound healing and general thriving (e.g., shiny fur, active behavior, grooming tendencies, etc.). Moisturizing skin care was carried out whenever deemed necessary. Mice were weighed daily (SCOUT STX2201, High-Performance Portable Balance) to monitor their recovery. To support a swift and pain-free recovery, mice were provided with soaked chow (buprenorphine drinking water) in the first 3 post-operative days and easily accessible dry chow on the cage bottom throughout the entire imaging period. The cages were equipped with modified enrichment to avoid mice getting stuck with the wings of the implanted AIW after surgery: tubes for tunnel handling with bigger diameter than regular paper rolls; the entry of paper houses was cut wider; long entangling nesting material was replaced with soft cotton buds and soft <1.5 cm short bedding material. Chewing sticks/balls were used for further enrichment. Mice were given 1-3 days to recover after surgery before the first imaging session.

#### Intravital labeling techniques and intravital cell identification

To label FDCs and thereby mark the light zone of GCs, mice were injected with 2 μg αCD35-iFluor647 in 100 μL sterile PBS i.v. 24 h before each imaging time point. αCD35 (clone 8C12, BD Biosciences 558768), was conjugated to iFluor647 (AAT Bioquest, Cat# 1031) using an in-house labeling protocol (final concentration 0.43 mg/mL and labeling density 12). Vasculature was visualized by injecting 1 mg 70 kDa FITC-dextran in sterile filtered PBS (100 μL of 10 mg/mL solution) via a heparinized (10 IU heparin/mL) tail vein catheter (total bolus injection volume 160 μL i.v.). T_REG_ and T_FR_ cells were visualized based on GFP expression of the fused DTR in FoxP3^+^ cells.^83^ T_FR_ cells were identified based on their localization in GCs.^50^ This included GFP^+^ cells localized within the FDC-labeled area, as well as areas in very close proximity, especially those close to TBMs. TBMs were easily identified based on bright vacuolar autofluorescence and their location close to FDC networks. Further microanatomical landmarks of follicles, such as perivascular-T (PT)-tracks,^61^ were visualized by large collagen bundles close to follicles, detected by second harmonic generation (SHG) imaging.^89^^,^^90^ The marginal zone was labeled only for fresh spleen explant imaging and was visualized by injecting 5 μg CD169-PE (142404; Biolegend) diluted in 100 μL PBS i.v. 10 min before euthanasia.

#### B cell harvest, purification and CFSE-labeling for adoptive B-cell transfer

Two C57BL/6 mice were euthanized by cervical dislocation. The spleen was surgically removed, placed in cold MACS buffer (DPBS, 2% FBS, 2 mM EDTA) and mechanically dissociated. The resulting single-cell suspension was filtered twice through 70 μm cell strainers pre-wetted with MACS buffer and centrifuged at 200 *g* for 10 min at 4 °C. Pelleted cells were resuspended in RBC lysis buffer (155 mM NH_4_Cl, 12 mM NaHCO_3_, 0.1 mM EDTA, pH 7.3) and incubated at RT for 3 min. Lysis was terminated by the addition of MACS buffer. Following filtration through a 70 μm cell strainer, cells were centrifuged at 200 *g* for 10 min at 4 °C and resuspended in 3 mL MACS buffer, after which cell counts were obtained using an automated cell counter (Nexcelom Cellometer K2) with ViaStain AOPI Staining Solution (Cat. No. CS2-0106-S). Splenocytes were subsequently incubated with 0.2 μL Fc Block (Rat Anti-Mouse CD16/CD32, Clone 2.4G2, BD Pharmingen, Cat. No. 553142) per 10^6^ cells for 5 min, whereupon a mixture of biotin-conjugated antibodies targeting CD43 (Ly-48), CD4 (L3T4), and Ter-119 was added (12 μL per 10^7^ cells, pre-diluted in 1 mL MACS). Cells were incubated for 30 min on ice, centrifuged at 200 *g* for 10 min at 4 °C, and resuspended in 1 mL MACS buffer, upon which magnetic anti-biotin microbeads (24 μL per 10^7^ cells) were added (B Cell Isolation Kit, Miltenyi Biotec, Cat. No. 130-090-862). After 20 min of incubation, stained splenocytes were filtered through a 70 μm strainer and B cells were purified using MACS LS columns (Miltenyi Biotec, Cat. No. 130-042-401), following the manufacturer’s protocol. This enabled negative selection of resting B cells, which were stored at −140 °C in medium containing 30% FBS and 10% DMSO (B cell freeze medium). The efficiency of the MACS purification was verified by flow cytometry.

On the day of the adoptive transfer, the negatively selected B cells were rapidly thawed in 9 mL pre-warmed Dulbecco’s Phosphate Buffered Saline (DPBS) (Biowest, Cat. No. X0515-500). Subsequently, cells were centrifuged at 280 *g* for 10 min at 4 °C and resuspended in sterile DPBS to achieve a concentration of 10^7^ cells/mL. In total, 8.86 × 10^6^ cells were thawed. A 500 μM stock solution of carboxyfluorescein diacetate succinimidyl ester (CFSE) (BD Horizon, Cat. No. 565082, Lot. No. 6312760) was prepared by dilution in sterile dimethyl sulfoxide (DMSO) (Sigma-Aldrich, Cat. No. D8418-50 ML). CFSE was added to the single-cell suspension to achieve a final concentration of 5 μM. Following incubation at 37 °C for 18 min in the dark, the cell suspension was diluted with nine volumes of B cell medium (RPMI-1640 medium supplemented with 10% FBS, 55 μM 2-mercaptoethanol, 1% penicillin/streptomycin, 10 mM HEPES, 1 mM sodium pyruvate, and 1% MEM NEAA). Finally, cells were centrifuged at 280 *g* for 10 min at 21 °C and resuspended in sterile Hanks' Balanced Salt Solution (HBSS) (Gibco, Cat. No. 14025092) to obtain a concentration of 10^7^ cells/mL. The final cell suspension had a concentration 1.06 × 10^7^ live cells/mL with a viability of 83.7%. 200 μL of the final cell suspension was injected into each mouse using a 30G insulin syringe.

#### Serial intravital two-photon microscopy

For intravital imaging, mice were anesthetized with isoflurane in medical air enriched with 33% oxygen (induction: 3.5% isoflurane; maintenance 0.7–1.5% isoflurane; flow rate: 0.6–1.2 L/min, using either SomnoSuite apparatus (Kent Scientific, United States) or Anesthetic Vaporizer UNO300VAP). After induction, mice received fluid therapy, 100 μL 0.9% NaCl s.c. and eyes were protected with eye ointment (Visc-ophtal øjengel 2 mg/g, Orifarm). The AIW was wiped with 70% ethanol and lens paper wrapped around a cotton swab. Then the mouse was placed on a heating plate and mounted in a custom 3D-printed holder for upright intravital imaging (Figure S7, adapted from Sardella et al;^42^). Primary imaging focus was found using epifluorescent light.

Microscopy was carried out with an Olympus FVMPE-RS multiphoton microscope with Fluoview FV31S software, equipped with an integrated 25× water immersion objective with an isolated tip and numerical aperture of 1.05 (Olympus, XLPLN25xWMP2: WD 2.00 mm), a MaiTai DeepSee Olympus laser (Spectra Physics), specifically tailored for the FVMPE-RS system, 2 high-performance multi-alkali PMTs and 2 GaAsP PMT detectors. Images were acquired in galvanometer scanning mode with two sequential excitation wavelengths, λ _Ex_ = 840 nm and λ _Ex_ = 940 nm, to capture distinct spectral features: λ _Ex_ = 840 nm was used to excite iFluor647 (CD35) and λ _Ex_ = 940 nm was used to excite GFP (FoxP3) and observe SHG (collagen). Fluorescent emission was collected in 4 channels (to balance signal to autofluorescence) in both excitation tracks using the following filter sets: Ch 1 & 2: 650/60 & 573/75, separated by Olympus FV30-SDM570 primary emission beam splitter from Ch 3 & 4 (GaAsP): 520/30 & 452/45. An IR short pass filter, Olympus BA685RXD, was used to only allow emission light below 685 nm.

Overview maps of the spleen area within the AIW FOV were manually outlined at a depth of around 120 μm below the capsule to set an imaging grid using the Multi-area Time Lapse (MATL) function in Fluoview to generate a 3D stitched overview of the entire imaging window during each ongoing imaging session. Single tiles of the 3D map were acquired in the MATL function at λ_Ex_ = 840 nm without averaging using 2 μs dwell time per pixel in 512 x 512 pixels resolution with 50 μm step size from 0 μm to 250 μm (0 μm marking the capsule) and 10% overlap of tiles. Individual GCs were acquired in volumetric image stacks with 5 μm step size between imaging frames, reaching a total depth of up to 220 μm – measuring from capsule to deepest imaging point. At λ_Ex_ = 840 nm, images were acquired in galvanometer scanning mode without averaging using 2 μs dwell time/pixel in 800 x 800 pixels resolution and at λ_Ex_ = 940 nm with 3 x line averaging using 4 μs dwell time per pixel in 800 x 800 pixels resolution. Detector Gains were adjusted in the beginning of each serial imaging cohort to balance fluorescence emission of interest and autofluorescence and then kept unchanged during each experiment. Laser Power was increased exponentially in depth to detect all fluorophores of interest throughout the entire 3D stack, while avoiding signal saturation, phototoxicity and bleaching.

For timelapse experiments, same hardware settings as described above were used. Images were acquired in one-way resonant scanning mode with 10x frame averaging in 512 x 512 pixels resolution at λ_Ex_ = 940 nm in 3 μm z-resolution and 1.5 – 4x Zoom. Timelapse movies were acquired for 5–45 min with a frame rate of 0.66–33 s, depending on the movie. Specific information of timelapse settings and respective frame rates can be found in video legends.

For PA-GFP experiments, same hardware settings as described above were used. Images were acquired in galvanometer scanning mode with 2 x line averaging using 4 μs dwell time/pixel in 512 x 512 pixels resolution at λ_Ex_ = 940 nm in 2 μm z-resolution. Image acquisition 1x before and multiple times over 2 weeks after PA-GFP activation. For PA-GFP activation small ROIs were set on trabeculae, vasculature wall or the capsule. ROIs were selectively photoactivated by one stimulation-scan at low laxer power for 0.5–2 s at λ_Ex_ = 800 nm. This protocol did not induce photodamage.

Each intravital two-photon imaging session lasted around 90–120 min per mouse. Appropriate respiration rate in anesthesia during image acquisition was monitored using an infrared camera (SVPRO 1080P Night Vision USB Camera CMOS OV2710 IR LED Infrared Webcam), core temperature was controlled using a rectal probe thermometer (Rodent thermometer BIO-TK8851, BiosebLab), and protective eye ointment was reapplied approximately every 30 min. After each imaging session, mice were rehydrated with another 100 μL 0.9% NaCl s.c. and provided with soaked chow. Mice usually woke up and started grooming themselves 2–5 min after anesthesia was withdrawn. Mice were imaged at 6 time points over 16 days.

#### Processing and analysis of intravital two-photon images

To compare volumetric changes in GCs in the spleen over time reliably, 3D imaging stacks from all six imaging days of individual GCs were standardized in stack position and stack size using FIJI (v2.14.0/154f).^91^ To standardize the imaging stacks, landmarks of capsule, vasculature and splenic trabeculae were manually matched to the same frame level over all imaging days. Based on matched frame positions, the entire 3D stack was reduced to the largest number of focal planes which contained identical biological structures throughout all imaging sessions. Standardizing imaging stacks also served as a checkpoint to reconfirm that the same GCs were observed over time. Each longitudinal imaging set was finally visualized with the Multi Stack Montage plugin from the PTBIOP Update Site^92^ to ensure consistency in stack standardization. Synchronization of windows is also possible with the built-in FIJI plugin “SyncWindows”.^91^

To visualize and analyze GCs in depth of the spleen, the imaging data was displayed in an orthogonal view using Reslice function, followed by z-projection in FIJI.^91^

To generate a quick, but reliable overview of the FDC-network size at respective imaging time points, an outline of the FDC-network was manually drawn and measured (area) on an *Average Z-Projection*, built-in Z function in FIJI^91^ of the λ _Ex_ = 840 nm track. Only frames with positive CD35 staining were selected for z-projection to avoid a distracting overlay of red pulp macrophages above the FDC network and facilitate visualization (Figure S3B). Before using the freehand tool to outline the FDC network, image noise was reduced by applying the “Despeckle” function in FIJI.^91^ Manual outline was eased by activating channel 1 (CD35^+^ and macrophages) and channel 3, while applying complementary pseudo colors (magenta and green) to both channels. Finally, the measured area was normalized by the measurement of the first observation timepoint.

Timelapse movies were preprocessed in FIJI^91^ and 4D reconstructed in Imaris v.8.2.1 for manual cell tracking using the surface tool. First, noise in ZT-hyperstacks was reduced by applying the “Despeckle” filter, then any 3D drift was corrected using the “Correct 3D Drift” plugin.^93^ For drift correction SHG-signal was used if not indicated otherwise. After drift correction, ZT-hyperstacks were further processed by applying a Gaussian Blur 3D (1 μm in all dimensions) and rolling ball background subtraction (rolling ball radius 50 pixels) and then imported in Imaris. Image preprocessing for T_FR_ cell visualization was carried out in FIJI without any additional plugins.^91^ For T_FR_ cell visualization, both excitation tracks were merged to eventually identify FoxP3^+^ (GFP^+^) cells in GCs. GC were identified based on their light zone with FDC labeling and the entire GC area was estimated based on TBM localization closely around the FDC network. Before merging channel 1 from λ _Ex_ = 840 nm track and channel 2–4 from λ _Ex_ = 940 nm track, background illumination from the respective channels was individually corrected by rolling ball background subtraction (rolling ball radius 50 pixels). Then, image noise of the newly merged image was reduced by applying the “Despeckle” filter. Lastly, the “Maximum 3D” filter was applied to further enhance the GFP-signal of single FoxP3^+^ cells (T_REG_ and T_FR_ cells) for better visualization.

Brightness and contrast of representative micrographs were linearly adjusted for each channel individually and any additional image processing is described in the respective figure legends.

#### Tissue harvest

For tissue harvest, mice were anesthetized with isoflurane and euthanized by decapitation. IngLN were removed and cleaned from surrounding fat tissue. Left (ipsilateral) and right (contralateral) IngLN from mice that underwent any kind of surgery, were kept separate. IngLNs were weighed on a precision scale (Mettler Toledo MS303S) and then either fixed in 4% w/v paraformaldehyde (PFA) for histological analyses or stored in FACS buffer (PBS, 2% heat-inactivated fetal calf serum (FCS), 1 mM ethylenediaminetetraacetic acid (EDTA)) on wet ice until tissue processing for flow cytometry. After 24 h of fixation, IngLN for histological analysis were transferred into 30% sucrose in PBS containing 0.1% sodium azide, incubated overnight, then thoroughly and gently cleaned with fine tweezers under a light microscope, embedded in OCT and frozen at −80°C until analysis. Spleens were carefully removed and either freshly prepared for immediate two-photon imaging or stored in ice-cold FACS buffer for flow cytometry (small piece). Spleens from AIW mice were taken out with attention to not damaging the parenchyma while separating the spleen from the glass coverslip. AIW-spleens were further divided into parts attached to the window (and subject to serial imaging) and parts that were distal to the AIW.

#### Flow cytometry

For flow cytometry, blood, spleen and IngLNs were processed following standard protocols: ca. 4 mm thick coronal spleen sections or IngLNs were mechanically macerated in 500 μL ice-cold FACS buffer by reusable pestles. Cell suspensions were then filtered through 100 μm cell strainers. Following filtration, spleen samples were centrifuged at 200 *g* at 4°C for 5 min. After supernatant removal, cells were resuspended in 400 μL red blood cell (RBC) lysis buffer (155 mM NH_4_Cl, 12 mM NaHCO_3_, and 0.1 mM EDTA) at room temperature for 5 min. The reaction was stopped with 1000 μL FACS buffer, then the suspension was centrifuged again at 200 *g* at 4°C for 5 min, the supernatant was removed, and the cells were resuspended in FACS buffer. All cell suspensions were stored on ice until plated.

Before adding 100 μL of cell suspension to wells, 20 μL of 1:50 diluted Fc-block (2.4G2), was added into wells of a 96-well plate. Antibodies and a viability dye were diluted in a 50:50 mix of FACS buffer and Brilliant Stain Buffer (BD Horizon) and 100 μL of the antibody mix was added to each plated sample and incubated for 30 min on ice. Then the plate was centrifuged at 200 *g* for 5 min 4°C, the supernatant was removed, and the pellet resuspended in 200 μL FACS buffer. The last step was repeated, and the samples were then freshly analyzed using either a BD LSRFortessa Cell Analyzer equipped with 4 lasers (405 nm, 488 nm, 561 nm, 640 nm) and 16 fluorescence detectors (BD Biosciences, San Jose, CA) or a Novocyte Quanteon 4025 equipped with 4 lasers (405 nm, 488 nm, 561 nm and 637 nm) and 25 fluorescence detectors (Agilent, Santa Clara, CA). Data were acquired in either BD FACSDiva Software version 8.0.2 (LSRFortessa, BD Biosciences, San Jose, CA) or NovoExpress version 1.6.2 (Quanteon, Agilent, Santa Clara, CA) and analyzed in FlowJo version 10.8.1 software (BD Lifesciences). Gating strategies can be found in Figure S5. Antibodies including dilutions used for flow cytometry experiments can be found in the key resources table.

#### Vibratome sections and two-photon imaging of freshly explanted thick spleen sections

Spleens for two-photon explant imaging were placed on ice and processed immediately after harvest. Using a vibrating blade microtome (Leica VT1200 S), 400–800 μm thick spleen slices (either transversal or coronal) were prepared. Beforehand, the vibratome tray was filled with wet ice and an object glass was mounted on the specimen plate with reusable sticky tack (leveled with a spirit level). Spleens were then briefly blot-dried on filter paper and then glued on the leveled object glass. Cutting parameters: speed: 0.18 mm/s; amplitude: 1 mm. Both spleen and blade were irrigated with PBS droplets during the entire cutting process and spleen slices were prevented from folding using a fine brush. Slices were mounted in PBS between two coverslips to allow imaging from both sides, and vacuum grease was used to seal the chamber. Slices were mostly imaged immediately but could however also be stored in the dark at 4°C overnight without an appreciable drop in imaging quality the next day.

Fresh explant two-photon microscopy was carried out on the same multiphoton system with similar hardware settings as during intravital microscopy. Images were acquired with two sequential excitation wavelengths, λ_Ex_ 840 nm and λ_Ex_ 940 nm, to capture distinct spectral features: λ_Ex_ 840 nm was used to excite iFluor647 and PE and λ_Ex_ 940 nm was used to excite GFP, PE and observe SHG. 3D image stacks were acquired using the MATL tile scan function in Fluoview (2x2 - 4x4 single tiles) to capture entire white pulp areas. Stack acquisition with 5 μm step size reaching a total depth of up to 200 μm (measuring from cut surface to deepest imaging point). All images were acquired in galvanometer scanning mode with 3 x line averaging, 4 μs dwell time/pixel at 800 x 800 pixels resolution. Detector Gains were adjusted in the beginning of each experimental cohort to balance fluorescent emission of interest and autofluorescence. Laser Power was exponentially increased in depth and adapted to avoid signal saturation and bleaching while still illuminating all fluorophores of interest throughout the entire 3D stack. Laser Power intensity was set lower than during comparable intravital scans.

#### Multi-staining and two-photon imaging of stained thick spleen sections

To visualize the biological dark zone of imaged GCs including the surrounding follicle and PALS, thick slices were stained with either Ki67-AlexaFluor488 (monoclonal (SolA15), eBioscience) only or IgD(*b*)-BV421 (BD; Clone: 217–270), CD3-AlexaFluor594 (BioLegend; Clone 17A2), and Ki67-AlexaFluor488 (monoclonal (SolA15), eBioscience) together subsequently to imaging. After imaging, slice orientation and approximate localization of imaged area were photo documented. Then, spleen slices were carefully removed from the imaging chamber and placed in an Eppendorf tube with 4% PFA overnight at 4°C. After fixation, slices were transferred into a tube with PBS and 0.1% sodium azide at 4°C until further permeabilization and staining (max. 1–2 months storage time). The slice that remained glued to the objective glass was occasionally imaged as well, and a fixation, storage and staining protocol for thick spleen sections glued to an objective glass was developed using 50 mL tubes, vacuum grease and custom 3D-printed humidified staining chambers. Prior to staining, spleen sections were washed in PBS (3 × 15 min) on a shaking table at room temperature in the dark. Samples were then incubated in the dark, overnight at room temperature in blocking buffer (5% FBS and 0.5% Triton X-100 in PBS with 0.1% sodium azide) on a shaker. The following day, the blocking solution was replaced with a staining buffer (2.5% FBS and 0.5% Triton X-100 in PBS with 0.1% sodium azide) containing Ki67-AlexaFluor488 (1:70). Samples were incubated for 48 h in the dark, at room temperature on a shaking table. After staining, sections were washed in PBS (3 × 15 min), then mounted between two coverslips using vacuum grease, in the same position as the fresh explant (with the help of the previous photo documentation to facilitate re-finding the same imaged areas). For multicolor staining, IgD(*b*)-BV421 (1:20) and CD3-AlexaFluor594 (1:70) were added first to the staining buffer and incubated with the spleen section for 48 h under the same conditions as described above. After 48 h incubation, Ki67-AlexaFluor488 (1:70) was added directly to the same staining solution without intermediate washing, and samples were incubated for an additional 48 h.

*FoxP3*-GFP signal disappeared in the staining process and was only visible in fresh spleen explants. Intravital labeling of αCD35-iFluor647 and αCD169-PE largely withstood the fixation and staining process and supported reidentification of imaged areas from fresh explants. Reidentification of the same areas was usually already achieved through oculars by reidentification of different CD169-PE staining patterns excited by epifluorescent light. Exact location was then confirmed with two-photon excitation. To facilitate reidentification of previously imaged areas, laser marks could additionally be set in fresh explants and re-found in stained slices (Figures S8A–S8C) as also described by Ritsma et al.^94^ Laser marks were set using λ_Ex_ 840 nm, 100 μs dwell time/pixel for 500 ms long per mark. The fragile consistency of fresh spleen tissue prevented a safe preservation of all set laser marks and hence multiple marks had to be set. The tissue shrank slightly during the fixation and staining process (Figures 6F–6I).

Images of stained spleen sections were acquired at λ_Ex_ 840 nm or λ_Ex_ 780 nm excitation. Large 3D image stacks were acquired using the MATL tile scan function in Fluoview in galvanometer scanning mode with 3-line averaging using 4 μs dwell time/pixel at 512 x 512 or 800 x 800 pixels resolution and stitched immediately post image acquisition with 10–30% overlap. Detector Gains were adjusted in the beginning of each experimental cohort to balance fluorescent emission of interest and autofluorescence. Laser Power was adjusted to avoid signal saturation and bleaching while still illuminating all fluorophores of interest throughout the entire 3D volume. Laser Power was increased exponentially in depth, but intensity was lower than during comparable intravital scans.

All representative images underwent image processing as described in figure legends. Additionally, representative images from explanted spleens both fresh and stained, were registered over another using the BigWarp plugin^95^ in FIJI for manual landmark-based image alignment (thin plate spline model). Autofluorescent macrophages (red pulp macrophages and TBMs) and CD169-PE labeling served as landmarks in all tracks.

#### Confocal microscopy and image analysis of inguinal lymph nodes

A Cryostar NX70 Cryostat (ThermoFisher) was used to cut 20 μm thick IngLN sections from the middle of each LN. Sections were mounted on SuperFrost+ glass slides (Epredia, REF J1800AMNZ). IngLN sections were acetone fixed (Merck, catalog number 1000141000). Briefly, the spleen samples were fixed in acetone for 10 min at room temperature. Primary conjugated antibodies were diluted in staining buffer (PBS, 2% v/v FBS, 0.1% w/v sodium azide) and spun for 10 min at 10,000 *g* at 4 °C to avoid aggregation. After fixation, slides were washed two times with PBS 0.1% w/v sodium azide and then stained overnight at 4°C in darkness. Slides were washed with staining buffer, and three times for 5 min with PBS 0.01% v/v Tween 20 spot-dried and mounted using Fluorescence Mounting Medium (S3023, Dako). After drying, slides were sealed airtight using nail polish and stored in darkness at 4°C until imaged. All imaging was done using a Zeiss LSM800 confocal microscope with a 10× objective. The following antibodies were used: CD35-iFluor647 (1/500 – in-house conjugation clone 8C12, BD Biosciences 558768 and AAT Bioquest, Cat# 1031), CD169-PE (1/500 – (142404; Biolegend)), and Thermo Fisher Scientific, S32354), Lyve-1-eFluor450 (1/300 – Invitrogen, clone: ALY7, 48-0443-82). Confocal images were processed and analyzed using FIJI (v2.14.0/154f).^91^ The total LN area was measured by manually outlining the entire LN in each image. All LN-lobes were included while surrounding lymphatic vessels and fat tissue were excluded. To quantify the area of intranodal lymphatic vasculature, the Lyve-1^+^ signal was analyzed within the manually defined LN boundary: First, the area outside the LN outline was cleared as described above. Then, an automatic thresholding (default mode) was applied to the Lyve-1 channel to generate a binary mask of the positive signal. To reduce background noise in the binary mask, a Gray Morphology Filter (radius = 1, shape = circle, operator = erode) was applied, followed by a median filter (Despeckle). The final area coverage of the Lyve-1+ mask within the LN was then measured and normalized to the previously measured LN size.

### Quantification and statistical analysis

#### Statistical analyses

Mice in different groups were randomly assigned, and studies were non-blinded by nature. A few animals and imaging datasets were excluded due to technical obstacles incl. bleeding and extreme tissue remodeling, respectively. Outliers were not excluded, and there was no statistical pre-determination of sample size. Statistical analyses were performed using GraphPad Prism (v. 10.1.1). QQ plots were used to determine normal distribution, the need for data transformation, and to choose a suitable statistical methodology. Statistical analysis was performed on raw data or on log-transformed data when QQ-plots and normality tests indicated log-normal distribution. Statistical significance was determined using two-tailed paired *t*-tests, and in case of grouped analyses, parametric or non-parametric tests depending on normality and homogeneity of variances (one-way ANOVA, two-way ANOVA or Kruskal-Wallis’s test). Data are expressed as means ± SD. *p*-values <0.05 were considered as significant and only *p*-values <0.05 are shown in graphs unless indicated otherwise. Test indicated in respective figure legend with n and *p*-values.

## Supplemental information

Document S1. Figures S1–S8

Video S1. Intravital timelapse movie of the spleen without and with 3D drift correction, related to Figure 2

Video S2. Serial intravital timelapse movie of a splenic follicle, related to Figure 2

Video S3. Intravital timelapse movie of a splenic blood vessel, related to Figure 2

Data S1. Custom Python script for nuclei counting using Cellpose on Google Colab, related to STAR Methods
