# Supplementary material for: Serial intravital microscopy reveals temporal dynamics of autoreactive germinal centers in the spleen
Source: iScience. 2026 Mar 11;29(4):115340. doi: 10.1016/j.isci.2026.115340 (PMC13059123; doi:10.1016/j.isci.2026.115340)
Supplement: Document S1. Figures S1–S8 [file mmc1.pdf]

iScience, Volume *29*

Supplemental information

Serial intravital microscopy reveals temporal dynamics of autoreactive germinal centers in the spleen

Layla Pohl, Thomas R. Wittenborn, Ali Shahrokhtash, Kristian S. Kastberg, Cecilia Fahlquist-Hagert, Lisbeth Jensen, Sofie Andkær Pedersen, Julia Karen Demtröder, Donato Sardella, Alain Pulfer, Duncan Sutherland, Santiago F. Gonzalez, Ina Maria Schiessl, and Søren E. Degn

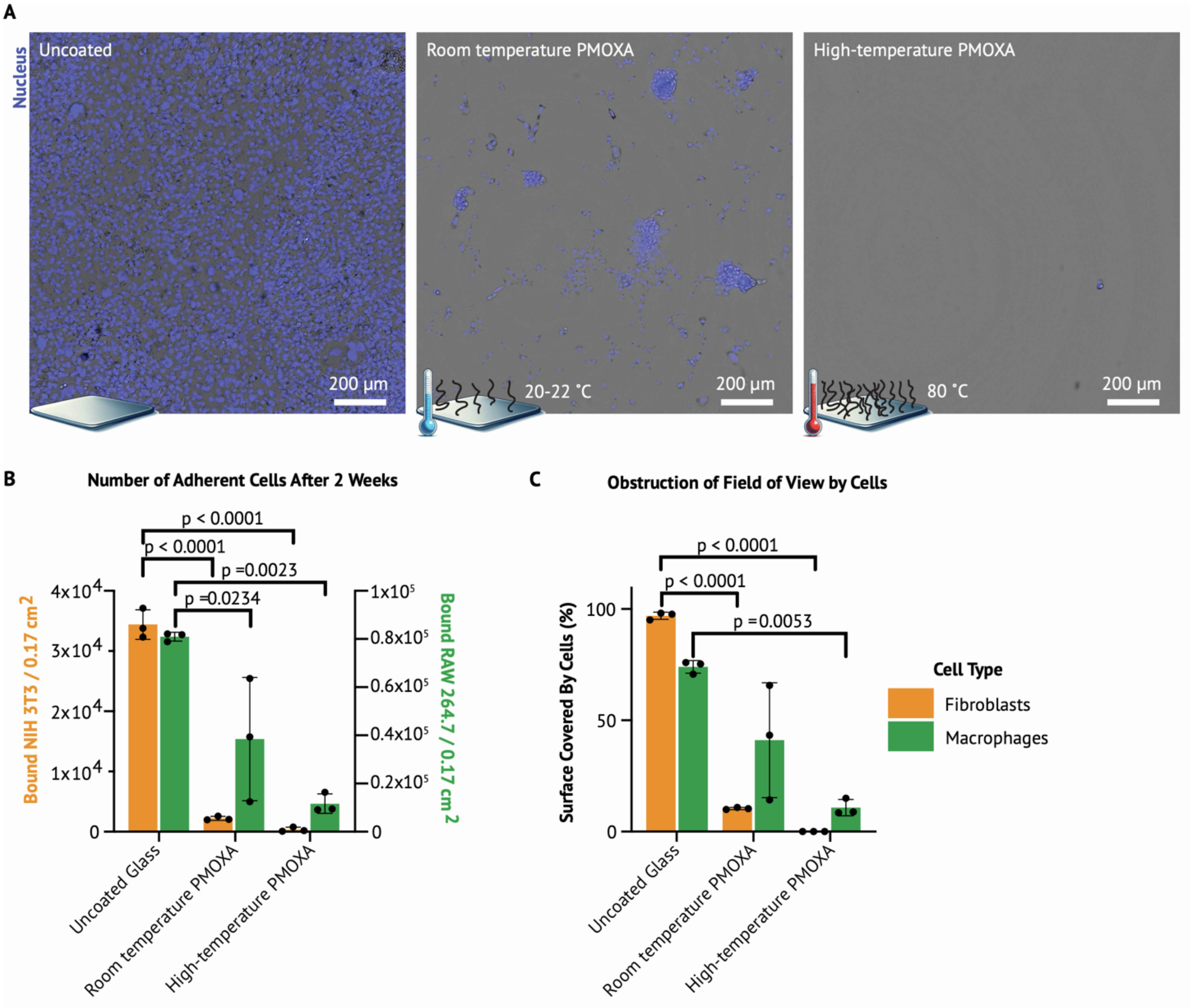

Figure S1. ***In vitro*** **comparison of different PMOXA passivating coatings after 2 weeks, related to Figure 1.** (A) Representative brightfield and nucleus-stained images of fibroblasts adhering and spreading on uncoated and passivated substrates after 2 weeks in serum-containing media. The uncoated substrates became overgrown within a few days, whereas room temperature PMOXA passivation significantly reduced non-specific cell adhesion, leading to the formation of 3D colonies on the few adherent cells. High-temperature PMOXA passivation almost entirely prevented fibroblast adhesion. (B) Quantification of the number of adherent cells for each passivation condition. High-temperature PMOXA passivation was significantly more effective in preventing non-specific adhesion of both fibroblasts and macrophages. (C) Quantification of surface area covered by cells on different substrates. High-temperature PMOXA passivation resulted in a clearer FOV due to a lower number of adhered cells and reduced cell spreading on the surface. Data are presented as means ± SD. P-values were calculated using ordinary one-way ANOVA, comparing each condition to the uncoated control (n = 3 with mean from 2 technical repeats).

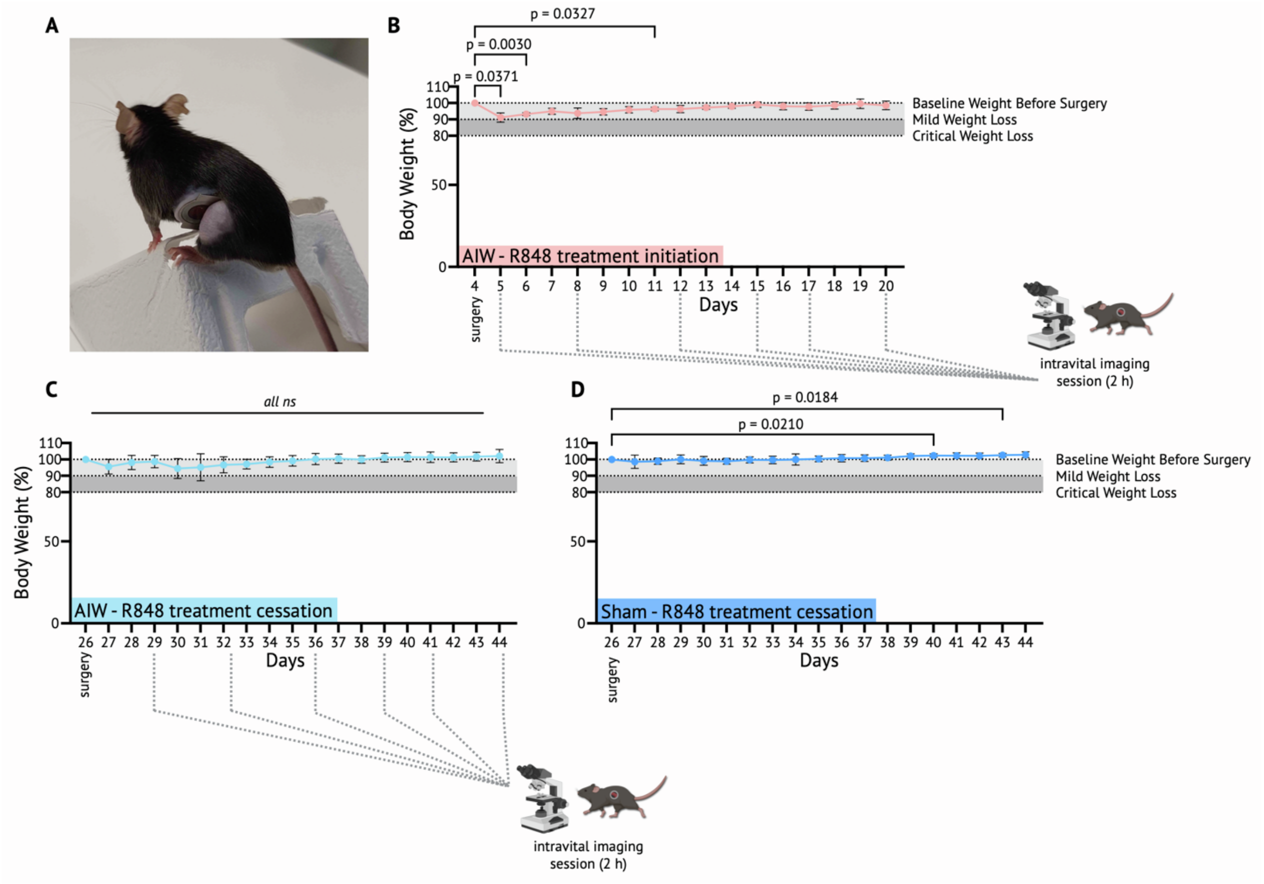

Figure S2. An AIW does not impact the thriving of mice, related to Figure 1.
(A) Mouse, 6 days after AIW implantation. The mouse is able to climb as usual when the AIW is correctly placed between spine, ribcage and hind leg. (B) Weight curves of mice with implanted AIW undergoing the R848 initiation treatment regimen (n = 4 from 2 cohorts). (C) Weight curves of mice with implanted AIW undergoing the R848 cessation treatment regimen (n = 8 from 5 cohorts). (D) Weight curves of sham operated mice without an implanted AIW undergoing the R848 cessation regimen (n = 6 from 3 cohorts). Bodyweight curves of mice were normalized to the bodyweight of each individual mouse on surgery day. The weight of the implanted AIW was subtracted from the measured bodyweight after AIW implantation. Light and dark grey areas indicate weight loss of 10% (mild) or 20% (critical), respectively. Below the grey areas, a weight loss would be indicated as critical, >20%, marking a humane endpoint. The baseline (100%) for bodyweight was set on surgery day before the surgical procedure for each mouse individually. Grey dotted lines indicate intravital imaging days. Data are represented as means ± SD. Adjusted P-values were computed with ordinary one-way ANOVA for multiple comparisons. Only P < 0.05 are indicated in the graph.

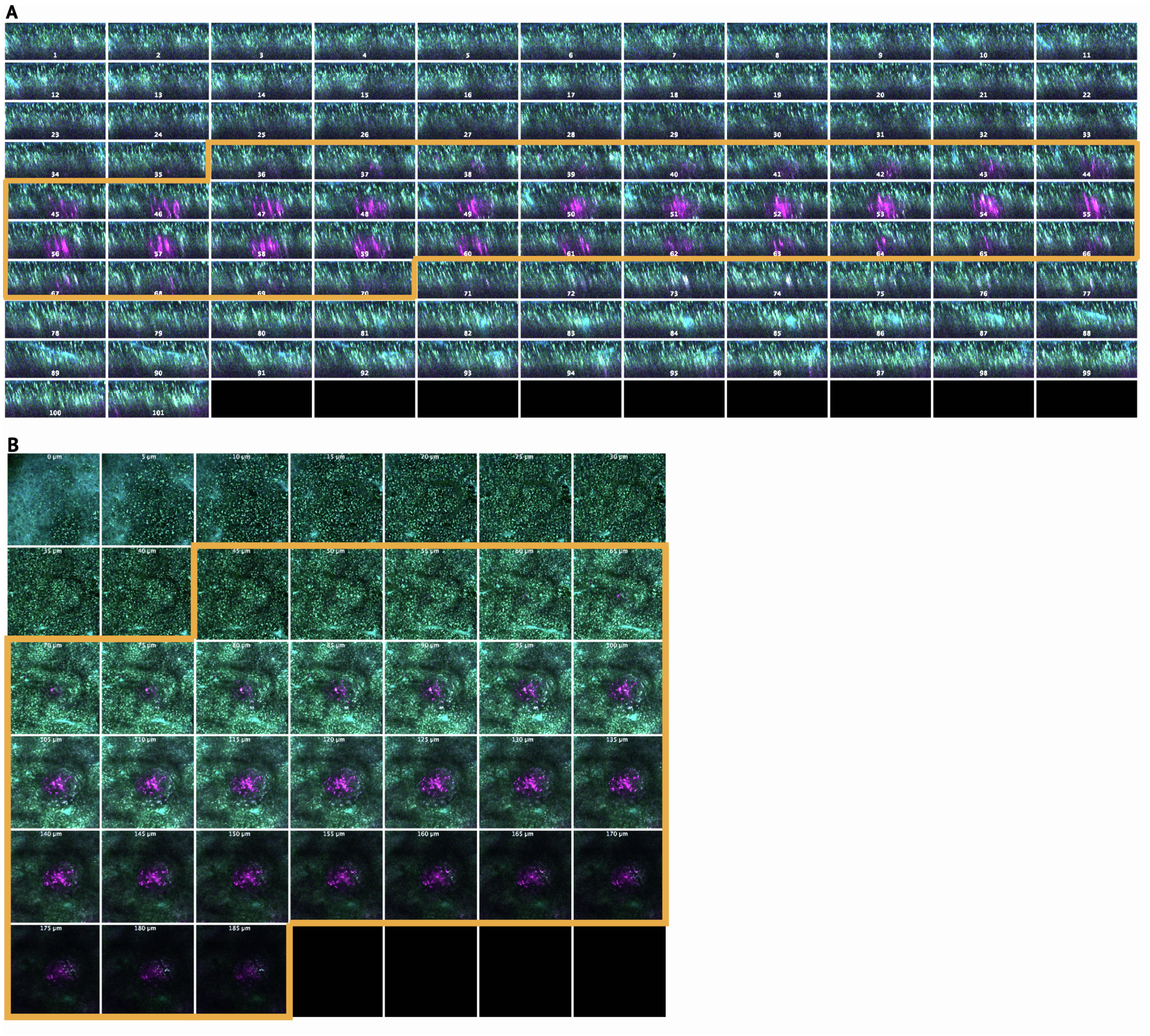

Figure S3. Y and Z projection of 3D datasets of the spleen to facilitate easy image display and analysis, related to Figure 2.

(A) Montage of all single Y-frames (orthogonally resliced) of the XZ-oriented imaging stack. Orange outline shows what frames were manually selected for 2D Y-projections. Only frames with staining/areas of interest were selected to be included in the Y-projection to avoid overlay of autofluorescent macrophages. (B) Montage of all single Z-frames of the XY-oriented imaging stack starting from capsule, 5 µm step size, until 185 µm below capsule. Orange outline shows what frames were manually selected for 2D Z-projections used for FDC network or GC remodeling analysis. Only frames with staining/areas of interest were selected to be included in the Z-projection to avoid overlay of autofluorescent macrophages.

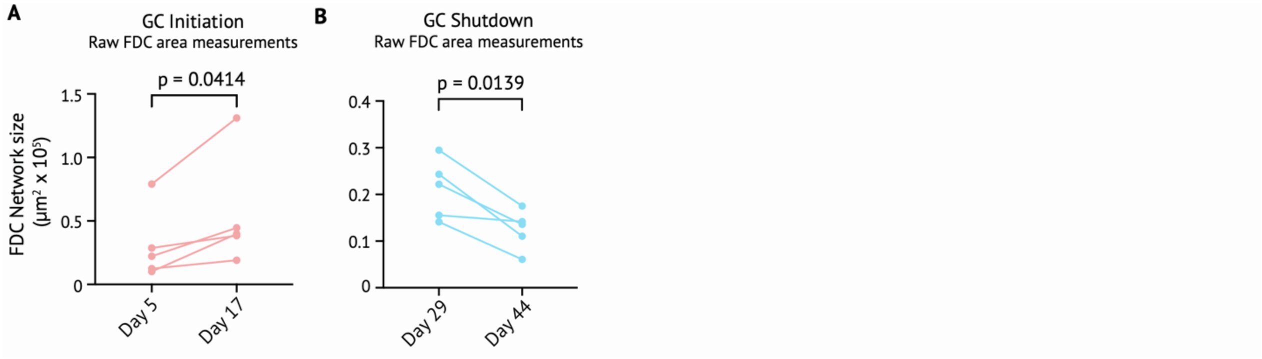

Figure S4. FDC Remodeling, related to Figure 4.
(A+B) Raw data of measured FDC area of a 2D Z-projection of imaging frames that were positive for CD35 labeling (compare with Figure S3B). Raw data is not normalized to the first observation day as in Figure 4C+D.

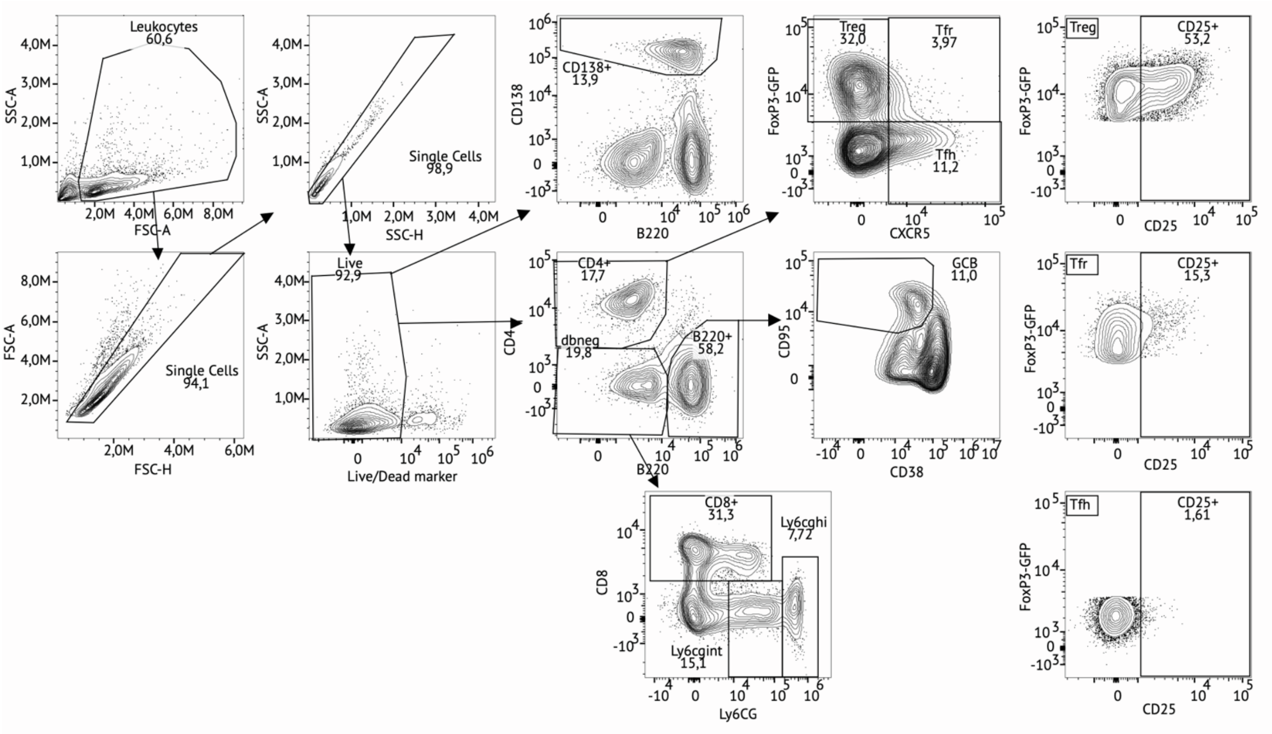

Figure S5. Flow Cytometry gating strategy, related to Figure 5.
Gating strategy of flow cytometry data, applied on spleen and IngLN datasets. Leukocytes were gated based on FSC-A and SSC-A, then singlet gating was applied using FSC height versus area and SSC height versus area, followed by live gating based on live/dead marker exclusion. From live, singlets, plasmablasts and plasma cells were gated as CD138. Similarly, from live, singlets, CD4+ T cells, B220+ B cells, and double negatives were gated. CD8+ cells, Ly6cg intermediate and high populations were sub-gated from the double negative gate. GCB cells were sub-gated from the B220+ B-cell population. Finally, CD4+ cells were gated as FoxP3+CXCR5- T_{REG}, FoxP3+CXCR5+ T_{FR}, and FoxP3-CXCR5+ T_{FH}. Each of the CD4+ sub-populations were assessed for CD25 positivity.

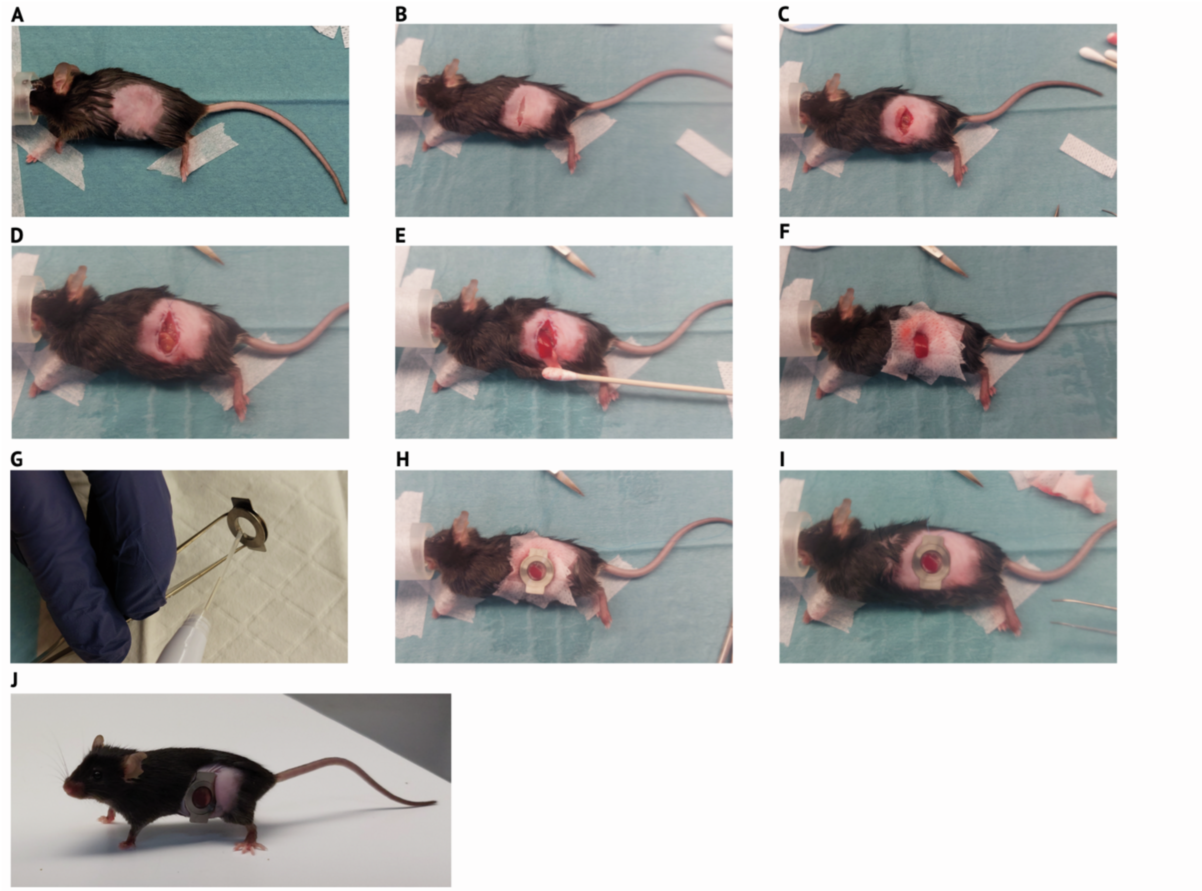

Figure S6. Surgical implantation of an abdominal imaging window (AIW) over the spleen, related to STAR Methods.
(A) Hair removal and skin disinfection. (B) Skin incision. (C) Incision of fat and muscle layer. (D) Setting purse string suture. (E) Gentle and careful spleen mobilization. (F) Skin protection with cotton tissue (to avoid glue touching the skin). (G) Adding glue on inner window border. (H) Placement of AIW with glue onto spleen. (I) Inserting skin-muscle layer into the rim of the window, followed by tightening and knotting of suture. (J) Awake mouse approx. 1 week after surgery.

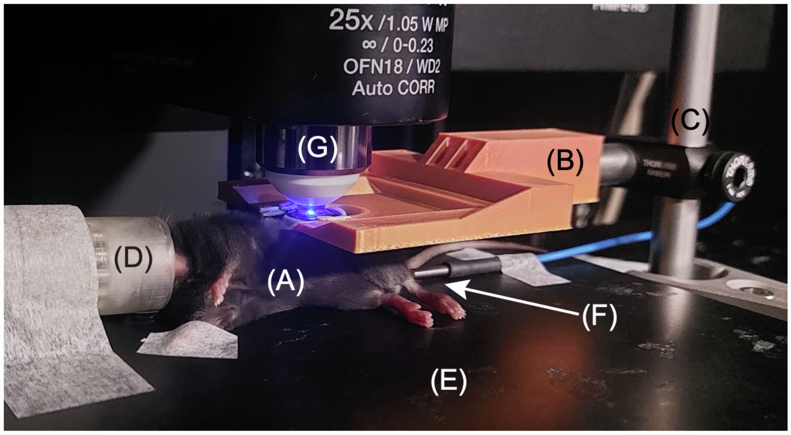

Figure S7. Intravital imaging setup with mouse in AIW holder, related to STAR Methods.
(A) Anesthetized mouse with implanted AIW. (B) Custom 3D-printed AIW holder. (C) Custom steel rod frame to stabilize the AIW holder. (D) Anesthesia mouthpiece (isoflurane, room air, and O_2. (E) Heating pad. (F) Rectal temperature probe to continuously monitor the core temperature of the mouse while imaging. (G) Two-photon objective with epifluorescent light.

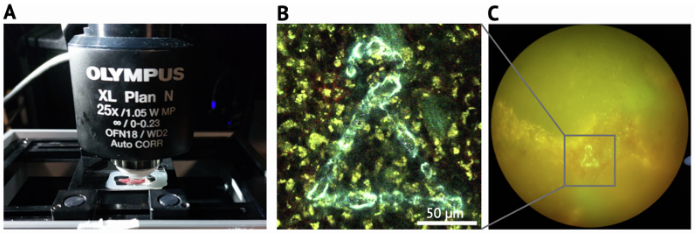

Figure S8. Laser marks in spleen explants for area re-identification after staining, related to Figure 6.
(A) Fresh thick spleen section under two-photon microscope. (B) Laser mark set in fresh spleen explants. Laser mark set and image acquired with two-photon microscope. Scale bar = 50 µm. (C) Laser mark identified through the ocular.
